# A Patient-Centered Website (Within Reach) to Foster Informed Decision-making About Upper Extremity Vascularized Composite Allotransplantation: Development and Usability Study

**DOI:** 10.2196/44144

**Published:** 2023-02-07

**Authors:** Karen B Vanterpool, Jessica Gacki-Smith, Brianna Kuramitsu, Max Downey, Michelle J Nordstrom, Michelle Luken, Tiffany Riggleman, Shannon Fichter, Withney Altema, James B Brucker, Carisa M Cooney, Gregory Dumanian, Sally Jensen, Macey Levan, Scott M Tintle, Gerald Brandacher, Elisa J Gordon

**Affiliations:** 1 Center for Surgical and Transplant Applied Research (C-STAR) NYU Langone Transplant Institute New York University Grossman School of Medicine Department of Surgery New York, NY United States; 2 Center for Health Services and Outcomes Research Northwestern University Feinberg School of Medicine Chicago, IL United States; 3 Uniformed Services University of the Health Sciences Center for Rehabilitation Sciences Research Walter Reed National Military Medical Center Bethesda, MD United States; 4 Henry M Jackson Foundation Bethesda, MD United States; 5 Department of Medical Social Sciences and Department of Surgery Northwestern University Feinberg School of Medicine Chicago, IL United States; 6 Department of Plastic and Reconstructive Surgery Johns Hopkins University Baltimore, MD United States; 7 Department of Orthopaedic Surgery Walter Reed National Military Medical Center Bethesda, MD United States; 8 Department of Surgery Vanderbilt University Medical Center Nashville, TN United States; 9 Center for Biomedical Ethics and Society Vanderbilt University Medical Center Nashville, TN United States

**Keywords:** hand transplantation, patient education, upper limb amputation, interviews, focus groups, disability, decision-making, accessible

## Abstract

**Background:**

Upper extremity (UE) vascularized composite allotransplantation (VCA; hand transplantation) is a reconstructive treatment option for patients with UE loss. Approximately 37 UE VCAs have been performed in the United States to date; thus, little is known about long-term psychosocial outcomes and whether the benefits outweigh the risks. To make an informed treatment decision, patients must understand the procedure, risks, and potential benefits of UE VCA. However, few educational resources are publicly available providing unbiased, comprehensive information about UE VCA.

**Objective:**

This paper described the development of a neutral, and accessible, educational website supporting informed decision-making about UE VCA as a treatment option for individuals with UE amputations.

**Methods:**

Website content development was informed by 9 focus groups conducted with individuals with UE amputations at 3 study sites. After initial website development, we conducted usability testing to identify ways to improve navigability, design, content, comprehension, and cultural sensitivity. Participants were administered the After-Scenario Questionnaire to assess user performance after completing navigational tasks, System Usability Scale to measure the perceived usability of the website, and Net Promoter Score to measure user satisfaction. Quantitative data were analyzed using descriptive statistics. Qualitative data were analyzed using rapid thematic analysis.

**Results:**

A total of 44 individuals with UE amputations participated in focus groups (n=37, 84%) and usability testing (n=14, 32%). Most participants in the focus groups and usability testing were male (24/37, 65% and 11/14, 79%, respectively) and White (27/37, 73% and 9/14, 64%, respectively), had unilateral limb loss (22/37, 59% and 12/14, 86%, respectively), and had mean ages of 48 (SD 9.2) and 50 (SD 12.0) years, respectively. Focus group results are organized into accessibility, website design, website development, website tone and values, sitemap, terminology, images and videos, and tables and graphics. Usability testing revealed that participants had a positive impression of the website. The mean After-Scenario Questionnaire score of 1.3 to 2.3 across task scenarios indicated high satisfaction with website usability, the mean System Usability Scale score of 88.9 indicated user satisfaction with website usability, and the mean Net Promoter Score of 9.6 indicated that users were enthusiastic and would likely refer individuals to the website.

**Conclusions:**

The findings suggest that our educational website, *Within Reach*, provides neutral, patient-centered information and may be a useful resource about UE VCA for individuals with UE amputations, their families, and health care professionals. Health care professionals may inform UE VCA candidates about *Within Reach* to supplement current VCA education processes. *Within Reach* serves as a resource about treatment options for patients preparing for scheduled or recovering from traumatic UE amputations. Future research should assess whether *Within Reach* improves knowledge about UE VCA and enhances informed decision-making about UE VCA as a treatment option.

## Introduction

### Background

Vascularized composite allotransplantation (VCA) is a reconstructive option that involves the transplantation of multiple tissues such as skin, muscle, bone, fat, nerves, and lymph nodes as a functional unit from primarily deceased donors [1,2]. Some examples of VCA include hand or upper limb, abdominal wall, face, uterus, and penis transplants. Upper extremity (UE) VCA has become a viable treatment option for people with UE loss (ie, hand or upper limb). Although UE VCA aims to and can potentially restore motor function and sensation and improve quality of life, this treatment poses risks, including lifelong immunosuppression, rejection, and long-term rehabilitation [3,4]. Episodes of acute rejection are common in VCA, and most VCA recipients experience rejection episodes; rates are higher in VCA than in solid organ transplants [5-7]. The function regained after UE VCA differs for each recipient. Regaining function requires several years of intensive rehabilitative therapy [8]. To date, approximately 150 UE VCAs have been performed worldwide, including 37 UE VCAs in the United States [9].

VCA raises many ethical issues, particularly regarding informed consent [10]. Informed consent concerns differ by VCA type given that the information to be disclosed about the risks, potential benefits, and alternatives differs, for example, for UEs, face, and uterus, as does the level of vulnerability that VCA candidates may experience [11-16]. In the context of UE VCA, a key ethical concern is that limited psychosocial data are available, which translates to limited information being publicly available. Thus, little is known about UE VCA recipients’ experiences with the informed consent process, which could inform the decision-making of individuals with UE loss regarding UE VCA [17]. The Organ Procurement and Transplant Network has not developed guidelines for information to be disclosed to UE VCA candidates, as it has for other organ transplants to ensure informed treatment decision-making. Consequently, the information provided by transplant programs to UE VCA candidates regarding the procedure likely varies. Such variation may contribute to people with UE amputations being inadequately informed and underprepared and feeling unduly pressured when considering this option. Educational materials (ie, websites, videos, and apps) can increase patients’ knowledge and improve patient-provider communication, decision-making, and informed consent [18-24].

However, few comprehensive educational resources exist regarding UE VCA as a treatment option. Although some UE VCA programs may provide information about this procedure on their institutional websites, their neutrality may not be apparent as they present either the positive or negative facets of this treatment option. Current VCA resources do not adequately educate the public [25]. Educational approaches are needed to inform patients about UE VCA; testimonials and videos showcasing the lived experiences of patients have been used to improve knowledge on various health topics, including living-donor kidney transplantation, HIV and AIDS, and maternal and child health [26-29]. No websites have been developed to provide balanced information intended to help individuals with UE amputations learn about UE VCA.

Extensive education is needed to prepare individuals with UE amputations to make informed treatment decisions regarding UE VCA because of the ethical complexity of this procedure. An increasing number of patients use the internet, specifically websites, as a source of information on health topics [30]. It is critical that web-based educational resources are usable to meet users’ needs, particularly in addressing user-encountered problems [31]. Mobile health educational resources (ie, mobile apps) that support patient decision-making about transplantation are considered highly acceptable and usable and should be used as a tool to improve patient education [32]. Patient-centered decision aids effectively improve knowledge of treatment options and are supported by patients [32,33].

### Objectives

We developed a neutral, patient-centered website, *Within Reach*. The website was designed to enhance understanding and promote transparency for those who are considering UE VCA as a treatment option to enable people with UE amputations to make informed treatment decisions. This paper describes the development and usability testing of *Within Reach*, the theoretical approaches guiding its development, and the data collection efforts.

## Methods

### Setting

The research team constituted a collaboration among 3 study sites: Northwestern University (NU), Johns Hopkins University (JHU), and Walter Reed National Military Medical Center (WR). Partners at NU affiliate Shirley Ryan AbilityLab and David Rotter Prosthetics collaborated. Data were collected from September 2021 to September 2022.

### Sample Population and Recruitment

Individuals eligible for participation included English-speaking adult (aged 18-65 years) civilians and military service members with an acquired unilateral or bilateral UE amputation, UE VCA *candidates* who contacted a transplant program to express an interest in UE VCA, UE VCA *participants* who provided consent for UE VCA evaluation, and UE VCA recipients. Individuals were excluded if they had a congenital UE amputation or an injury that caused severe nerve damage to the residual limb or neurological damage that would disqualify them from UE VCA.

Eligible individuals were recruited through collaborating sites or through community and online support groups for individuals with UE amputations. Each research site mailed or emailed introductory letters to all potentially eligible participants followed by a phone call 1 week later to assess interest in participation. Patients in support groups were recruited through listserves, emails, or web-based postings advertising the study using a flyer; interested individuals contacted the study team directly.

### Ethics Approval

The institutional review boards at NU (STU00209718), JHU (00225728), and WR (WR-EDO-2020-0432, relying on the NU institutional review board) approved this study. The US Army Medical Research and Development Command Human Research Protection Office approved this study at NU (E00798.1a), JHU (E00800.1a, E00799.1a), and WR (E00801.1a). Verbal informed consent was obtained from potential participants before enrollment in the study. Participants were compensated with US $35 for their time.

### Website Development Process

The website is intended to be used by individuals with hand and upper limb amputations, their families, and their health care providers. This study used a cross-sectional approach for qualitative data collection involving telephone and web-based focus groups. We supplemented data collection with mixed methods research for refinement and usability testing of educational materials. This information enabled the elaboration and clarification of the findings, increasing the validity of the results, and informed subsequent data collection [34,35].

Website development was guided by the Health On the Net Foundation code of conduct certification guidelines, which provide credibility that the website follows a code of ethics ensuring that it provides quality information [36]. The design of *Within Reach* followed a six-step website development process: (1) discovery phase, (2) planning, (3) design, (4) development, (5) launching the website, and (6) maintenance [37] (Textbox 1).

The selection of medical content for the website was guided by elements of informed consent, including the risks, benefits, procedures, alternatives, and voluntary nature of UE VCA. In addition, content was driven by a review of the literature; conversations with health care providers working in UE VCA (eg, UE VCA clinicians or surgeons, hand reconstructive surgeons, and occupational therapists); in-depth and semistructured interviews with individuals with UE loss and UE VCA candidates, participants, and recipients about their information needs regarding UE VCA [38]; and focus groups, which provided input on the website sitemap and topic and subtopic headers. Participants suggested topics that should be included or removed. Our study team, comprised of clinicians (ie, 2 hand surgeons, 1 UE VCA surgeon, 3 occupational therapists, and 1 VCA clinical researcher), 1 social worker, 3 social scientists, 2 ethicists, 1 instructional designer, and >10 research staff, provided feedback on several drafts of the website content. The research team consulted with the clinicians through multiple iterations of content development for clarification and refinement.

**The 6-step website development process.**

**Discovery phase**

- The discovery phase entailed determining the mission, goals, target audience, and content of the website. The website is intended to be a patient-centered resource for health information about upper extremity vascularized composite allotransplantation (VCA). The website is Americans with Disabilities Act–compliant, which includes the use of UserWay (UserWay Inc), an accessibility plug-in that increases Web Content Accessibility Guidelines 2.0 compliance. The content and design of the website were derived from published data on upper extremity VCA interviews and focus groups with members of the target audience.

**Planning**

- Planning involved developing a website sitemap and a list of topics and subtopics. The sitemap guided the site’s development of content and the navigational system. The website provides information about VCA, including steps in the evaluation process, factors to consider during decision-making, risks of surgery, rehabilitation process, alternative treatment options, and resources.

**Design**

- The design process required determining the appearance of the site. The research team provided the website developer with design examples of health and educational websites that the team preferred. Mock-ups were developed iteratively and jointly by the research team and the website developer based on feedback from focus group participants to ensure that the content had face validity. The content was revised when several participants suggested changes, areas arose as problematic, or the rationale for change was sound. VCA clinicians provided input on the accuracy of the website content.

**Development**

- The development process required creating the actual functional website. The website used quality assurance strategies (ie, the 16-item validated *Quality Assurance Rating Tool for Internet Health Sites*) [39] and web 2.0 design comprising the integration of many information sources and live updates of information.

**Launching the website**

- Testing and delivery involved testing the functionality of the website to ensure that there were no broken links. The website developer ensured that the website was compatible with the Americans with Disabilities Act requirements. The website underwent a final test to ensure that all aspects were functioning correctly before launching it.

**Maintenance**

- Maintenance entails a long-term strategy to update information on the website. The research team collaborated in updating and editing the website during its development. When the study was completed, the site URL was posted on the American Society for Reconstructive Transplantation server.

### Structure

**Figure 1:**
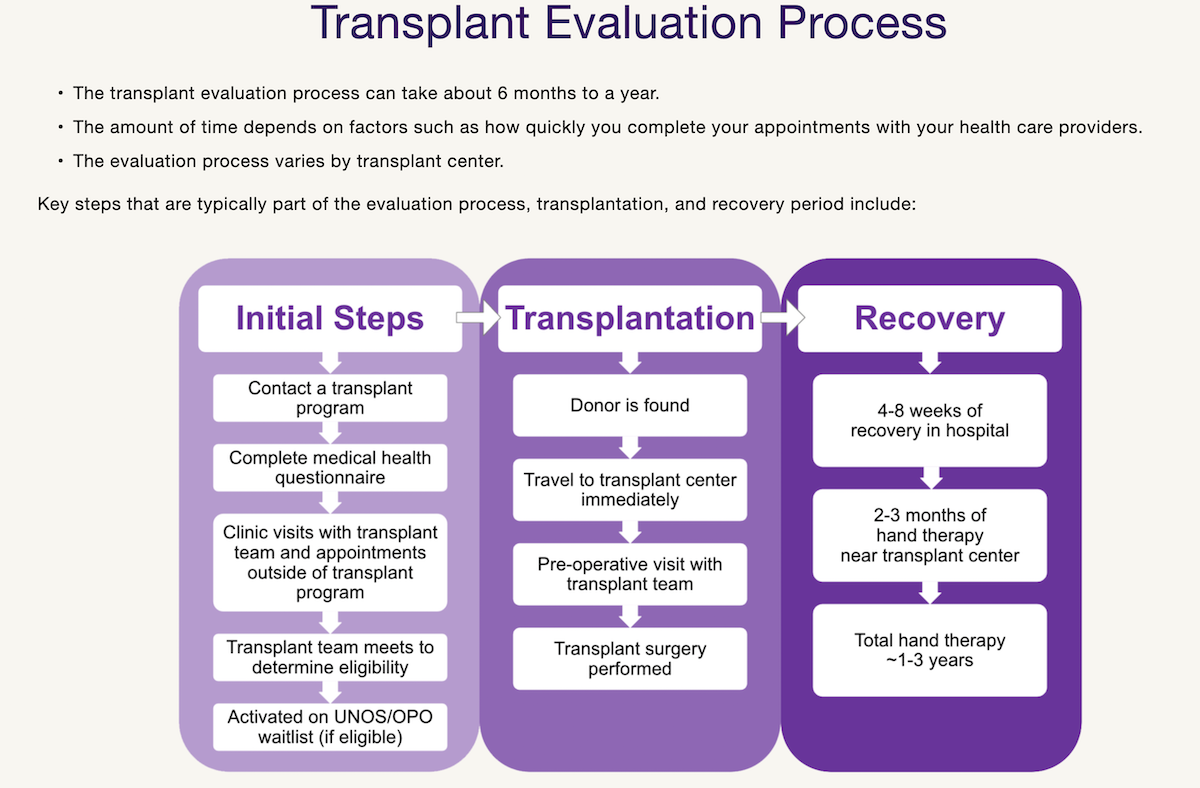
This screenshot illustrates the steps in the upper extremity vascularized composite allotransplantation evaluation process. OPO: organ procurement organization; UNOS: United Network for Organ Sharing.

*Within Reach* has eight main sections: (1) *Hand/Arm Transplant* describes background information on VCA, functional outcomes, psychological outcomes, patient experience, potential benefits, myths and facts, and limitations; (2) *Process* covers patient eligibility, characteristics of a good candidate for the procedure, evaluation process, transplant team, contacting a transplant center, postevaluation process, and surgical process (Figure 1); (3) *Risks* presents surgical and medical risks, acute and chronic rejection, removal of the hand or arm, antirejection medication, pain and discomfort, and emotional and social difficulties; (4) *Recovery* includes the surgical recovery and rehabilitation process, lifestyle changes, and the caregiver’s role; (5) *Options* covers information on cosmetic, body-powered, myoelectric, and electric prostheses as well as switch and pressure sensors, LUKE arm, osseointegration, and the option of no prostheses; (6) *Decision-Making* focuses on factors to consider when deciding whether to pursue UE VCA, including treatment options, weighing treatment options, impact of UE VCA transplant on mental health, social support, long-term commitment of UE VCA, and the financial costs of UE VCA; (7) *Resources* includes a list of transplant centers performing UE VCA, a library of all videos from the website, a downloadable question prompt sheet to guide patient discussions with providers, a downloadable pamphlet about the website that health care professionals can disseminate to patients, a list of websites or online support groups, and a list of our journal publications and paper presentations; and (8) *About Us* describes the professional backgrounds of the research team and lists the organizations that collaborated to develop the website.

### Theoretical Frameworks

The theoretical frameworks that guided website development to enable learning were Social Cognitive Theory by Bandura [40] and the conditions of learning by Gagne et al [41]. Both theories have been used to guide the development of health websites for diverse populations [26]. The conditions of learning by Gagne et al [41] identify 9 instructional design strategies that educational resources should use to increase the likelihood of learning (eg, gaining attention, stimulating recall of prerequisite learning, and presenting the stimulus material). Social Cognitive Theory by Bandura [40] posits that observational learning occurs by observing others and modeling behavior. The ability to model behavior is related to self-efficacy, which is an individual’s belief in their ability to reproduce an observed behavior [42]. Social Cognitive Theory was applied to *Within Reach* by including UE VCA recipient video testimonials for observational learning (modeling).

### Data Collection Activities

#### Focus Groups

We conducted 9 focus groups (n=3, 33% per site) via teleconference or videoconference. Focus groups were conducted to gather user preferences on website wireframe (ie, blueprint) concepts, content, design, and functionality regarding the user-centered design [43]. Focus groups initially assessed preferences regarding the website name, logo, photographs, website design, sitemap, headers, terminology, and content (ie, mission statement, myths or facts, quotes, and data tables and graphics). Thereafter, focus groups entailed formative testing involving showing initial website sections (eg, decision-making, risks, recovery, and resources) to obtain feedback on content, design, graphics, tables, and images. Focus groups were staggered across study sites to allow the team to debrief and identify website modifications so that feedback could be obtained on the revised iterations of the website content and graphics in subsequent focus groups. Focus groups were facilitated by a single researcher at each site (EG, MD, and MN).

Participants were asked what they liked and disliked, their perceptions of the website’s cultural sensitivity to the community of individuals with UE amputations, and how they would improve website content [44]. The focus groups were audio recorded and lasted approximately 2 hours. After each focus group, participants completed a web-based survey assessing sociodemographics (eg, age and sex), health literacy (“How often do you need someone to help you when you read instructions, pamphlets, or other written material from your doctor or pharmacy?” anchored by “Never” and “Always”; response options of “Never” and “Rarely” were reflected as adequate health literacy) [45], and perceptions of the website. Perceptions of the website were assessed using a 5-point Likert scale from 1 (strongly agree) to 5 (strongly disagree). Multimedia Appendix 1 provides the focus group moderator guide, and Multimedia Appendix 2 describes the website materials presented and associated questions about the website.

#### Usability Testing

Usability testing was conducted at 2 sites (NU and WR) to assess user experience with *Within Reach*; testing focused on the ability of users to complete specific tasks [46]. Usability testing provided user feedback on “the individual’s entire interaction with the thing [*Within Reach*], as well as the thoughts, feelings, and perceptions that result from that interaction” [46], which informed the refinement of the website.

Usability testing was conducted in person and via videoconference. During usability testing, the website was viewed exclusively on laptops provided by the research staff in person or on computers owned by the study participant at each site via videoconferencing. The website was not trialed on a phone or tablet to maintain the consistency and reliability of the results as the website configuration differs slightly depending on whether it is accessed via laptop or mobile phone.

The research staff observed each participant as they freely navigated the website and then completed 5 task scenarios. The task scenarios required participants to find specific information on the website while speaking aloud to convey the thought processes informing their decision to use a certain navigation route. This activity revealed which website sections needed content or design modifications to improve website navigability. The research team tracked website usability metrics (eg, time needed to find sections and satisfaction with navigation and content).

For each task scenario, participants were asked the following: “How easy was it to find what you were looking for?” which assessed information findability on a Likert scale (range 1-5), and “How satisfied are you with the information presented in this section?” which assessed information satisfaction on a Likert scale (range 1-5). Participants completed the After-Scenario Questionnaire (ASQ), a 3-item, 7-point Likert scale anchored by 1=“strongly agree” to 7=“strongly disagree” assessing satisfaction with the usability of the website based on each task scenario [47]. A lower score indicated higher level of satisfaction with usability.

Upon completing the task scenarios, participants were asked standardized survey questions to determine website usability. Participants completed the System Usability Scale (SUS), a 10-item, 5-point Likert scale that assessed the usability of the website (Cronbach α=.85) [48]. Response options ranged from 1=“strongly disagree” to 5=“strongly agree.” Scores ranged from 0 to 100; higher scores represented greater website usability [49,50]. Participants completed the Net Promoter Score (NPS), an 11-point scale that assesses a user’s loyalty to the website by asking, “How likely are you to recommend this website?” Scores range from 0 to 10; higher scores represented greater enthusiasm for the website. Participants were asked, “Rate your overall experience with this website,” assessing overall website experience on a Likert scale (range 1-7). Higher scores represented greater satisfaction. Perceptions about the website being culturally sensitive to individuals with upper limb amputations and to racial or ethnic minorities were assessed using nine 5-point Likert scale items [51,52]. Usability testing was audio recorded and averaged 92 (SD 30.7; range 52-158) minutes.

### Statistical Analysis

Descriptive statistics were used on the post–focus group survey items and usability testing items assessing attitudes toward UE VCA, satisfaction with the website, ASQ scores, SUS scores, NPS scores, perceptions of cultural sensitivity, and demographics. We calculated frequencies, means, and SDs. Analyses were performed using SPSS Statistics (version 27; IBM Corp).

### Qualitative Analysis

A rapid thematic analysis was applied to obtain feedback on the *Within Reach* website. The focus groups were analyzed using a rapid-cycle evaluation approach [53]. This approach allowed the research team to quickly assess the focus group data to make critical decisions and improve the quality of the website as it was developed [54]. The process entailed the following rapid analysis steps: (1) creating a neutral domain or code name to correspond to focus group questions (eg, website name, terminology preferences, and general feedback), (2) creating a template to identify and organize key points under each domain or code, and (3) summarizing focus group findings at each site using the template to highlight the key points that emerged under each domain or code. The completed template was then reviewed and edited by a second research team member at each site. In total, 2 team members per site independently reviewed the audio recordings and notes from the focus groups to complete the template and (4) consolidated key findings across templates into analytic matrices organized by domain or code. The matrices organized data across focus groups and study sites by domain or code to facilitate the identification of similarities and differences within a domain or code across sites and focus groups.

## Results

### Focus Groups

#### Demographics

Of the 138 eligible participants contacted, 37 (26.8% participation rate) enrolled in the focus groups (Table 1). Of the 37 participants enrolled in the focus groups, 12 (32%) were from NU, 14 (38%) were from JHU, and 11 (30%) were from WR. The mean age of the participants was 48.3 years. Most participants were male (24/37, 65%) and White (27/37, 73%), had adequate health literacy (35/37, 95%), and had undergone unilateral amputation (22/37, 59%). The mean number of years since the first amputation was 13 (SD 13.1; range <1-53) years.

**Table 1:** Focus group participants’ sociodemographic characteristics (n=37).

Characteristics			Total		NU^a^ (n=12)		JHU^b^ (n=14)		WR^c^ (n=11)
Age (years), mean (SD; range)			48.3 (9.2; 32-66)		52.7 (5.8; 42-60)		45.6 (10.9; 32-64)		47 (8.7; 35-66)
**Sex, n (%)**									
	Male	24 (65)		8 (67)		8 (57)		8 (73)	
	Female	13 (35)		4 (33)		6 (43)		3 (27)	
**Ethnicity^d^, n (%)**									
	Not Hispanic or Latino	34 (92)		12 (100)		13 (93)		9 (82)	
	Hispanic or Latino	2 (5)		0 (0)		0 (0)		2 (18)	
**Race^e^, n (%)**									
	White	27 (73)		10 (83)		12 (86)		5 (45)	
	Black or African American	6 (16)		1 (8)		2 (14)		3 (27)	
	Other	4 (11)		1 (8)		0 (0)		3 (27)	
**Marital status, n (%)**									
	Married or domestic partner or civil union	23 (62)		7 (58)		7 (50)		9 (82)	
	Separated or divorced	9 (24)		4 (33)		3 (21)		2 (18)	
	Never married or single	5 (14)		1 (8)		4 (29)		0 (0)	
**Education, n (%)**									
	High school graduate	6 (16)		2 (17)		2 (14)		2 (18)	
	Some college	8 (22)		2 (17)		3 (21)		3 (27)	
	College graduate	15 (41)		5 (42)		5 (36)		5 (45)	
	Postgraduate degree	8 (22)		3 (25)		4 (29)		1 (9)	
Health literacy (adequate), n (%)			35 (95)		11 (92)		12 (86)		8 (73)
**Employment status, n (%)**									
	Employed full time	12 (32)		6 (50)		3 (21)		3 (27)	
	Employed part time	2 (5)		0 (0)		2 (14)		0 (0)	
	Not employed	2 (5)		0 (0)		2 (14)		0 (0)	
	Homemaker	2 (5)		0 (0)		1 (7)		1 (9)	
	Student	1 (3)		0 (0)		0 (0)		1 (9)	
	Disabled	7 (19)		3 (25)		3 (21)		1 (9)	
	Retired	11 (30)		3 (25)		3 (21)		5 (45)	
**Income (US $), n (%)**									
	<15,000	2 (5)		2 (17)		1 (7)		1 (9)	
	Between 15,000 and 34,999	5 (14)		0 (0)		3 (21)		0 (0)	
	Between 35,000 and 54,999	2 (5)		1 (8)		1 (7)		0 (0)	
	Between 55,000 and 74,999	4 (11)		3 (25)		1 (7)		0 (0)	
	Between 75,000 and 94,999	8 (22)		0 (0)		3 (21)		5 (45)	
	>95,000	10 (27)		5 (42)		2 (14)		3 (27)	
	Prefer not to answer	6 (16)		1 (8)		3 (21)		2 (18)	
**Primary health insurance^f^, n (%)**									
	Private	13 (35)		5 (42)		6 (43)		2 (18)	
	Medicaid or Medicare	19 (51)		6 (50)		8 (57)		5 (45)	
	Tricare	10 (27)		0 (0)		2 (14)		8 (73)	
	Other	4 (11)		1 (8)		1 (7)		2 (18)	
**Health status^d^, n (%)**									
	Excellent	5 (14)		1 (8)		3 (21)		1 (9)	
	Very good	15 (41)		6 (50)		4 (29)		5 (45)	
	Good	12 (32)		4 (33)		5 (36)		3 (27)	
	Fair	4 (11)		1 (8)		2 (14)		1 (9)	
	Poor	N/A^g^		N/A		N/A		N/A	
**Dominant hand amputated^d^, n (%)**									
	Yes	24 (65)		9 (75)		7 (50)		8 (73)	
	No	11 (30)		3 (25)		5 (36)		3 (27)	
**UE^h^ amputated, n (%)**									
	Right	12 (32)		4 (33)		2 (14)		6 (55)	
	Left	10 (27)		3 (25)		6 (43)		1 (9)	
	Both	13 (35)		5 (42)		4 (29)		4 (36)	
	Prefer not to answer	2 (5)		0 (0)		2 (14)		0 (0)	
**Amputation type, n (%)**									
	Unilateral	22 (59)		7 (58)		8 (57)		7 (64)	
	Bilateral	13 (35)		5 (42)		4 (29)		4 (36)	
	Prefer not to answer	2 (5)		0 (0)		2 (14)		N/A	
**Amputation level, n (%)**									
	Below elbow	22 (59)		9 (75)		6 (43)		7 (64)	
	Above elbow	15 (41)		3 (25)		8 (57)		4 (36)	
**Current prosthesis type^i^, n (%)**									
	Cosmetic	2 (5)		0 (0)		1 (7)		1 (9)	
	Mechanic	15 (41)		7 (58)		2 (14)		6 (55)	
	Myoelectric	16 (43)		3 (25)		6 (43)		7 (64)	
	None	10 (27)		3 (25)		6 (43)		1 (9)	
**Time since the last amputation (years)^j^, n (%)**									
	<1	1 (3)		0 (0)		1 (7)		0 (0)	
	1-2	6 (16)		1 (8)		4 (29)		1 (9)	
	3-5	6 (16)		3 (25)		3 (21)		0 (0)	
	6-9	5 (14)		2 (17)		2 (14)		1 (9)	
	10-15	10 (27)		3 (25)		3 (21)		4 (36)	
	16-25	3 (8)		1 (8)		0 (0)		2 (18)	
	>25	6 (16)		2 (17)		1 (7)		3 (27)	
**Type of participant, n (%)**									
	Person with UE amputation	30 (81)		11 (92)		8 (57)		11 (100)	
	VCA^k^ candidate or participant	5 (14)		1 (8)		4 (29)		0 (0)	
	VCA recipient	2 (5)		0 (0)		2 (14)		0 (0)	

^a^NU: Northwestern University.

^b^JHU: Johns Hopkins University.

^c^WR: Walter Reed National Military Medical Center.

^d^Percentages do not add up to 100 as some participants did not respond.

^e^“Other” included people who identified as American Indian or Alaska Native (1/4, 25%), Asian (1/4, 25%), Native Hawaiian or other Pacific Islander (1/4, 25%), or Malagasy (1/4, 25%).

^f^Percentages do not add up to 100 as some participants had multiple forms of insurance.

^g^N/A: not applicable.

^h^UE: upper extremity.

^i^Percentages do not add up to 100 as some participants were using multiple prostheses.

^j^Some participants had multiple surgeries for their amputation or multiple amputations.

^k^VCA: vascularized composite allotransplantation.

#### Accessibility

The website content and design entailed addressing the unique challenges that individuals with a UE amputation may encounter when using the website (Table 2). Focus group participants perceived tiles to be easier to navigate compared with scrolling through drop-down lists to access information. Participants mentioned that tiles would enable individuals with recent UE amputations who may be using their nose or a stylus to navigate the website more easily. Large tiles for each topic area were incorporated into the website layout. A search button or function was added to enable individuals with UE amputations to use voice commands to navigate through the website. Voice-over capability was added to some of the images and graphics to improve the experience for individuals who may be challenged navigating the website.

**Table 2:** Website changes based on focus group feedback.

Design element and subcategory			Original version		Final version
**Accessibility**					
	Tiles	Links to each subsection of the corresponding section		A matrix of tiles for each subsection provides a larger surface area for users to click on the desired subsection.	
	Search button	Absent		Users could search for specific keywords and be taken directly to relevant sections of the website.	
	Voice-over	Absent		Recorded narrative descriptions of figures and graphs.	
**Website design** **(to satisfy the conditions of learning by Gagne et al [41])**					
	Gaining attention	Short, interesting factoids about the history, outcomes, and process, among other things, of UE^a^ VCA^b^ were included in a list of questions called “Did You Know?” to gain the attention of users (eg, “Did you know that the first UE VCA was performed in 1999?”).		No change	
	Informing the learner of the objective	Each of the 8 main sections of the website starts with 1-2 sentences explaining the objective of that section.		No change	
	Stimulating recall of previous learning	The “Myths and Facts” subsection under the “Hand/Arm Transplant” section allows for knowledge application by testing the user’s accurate knowledge of a topic.		No change	
	Presenting the stimulus or content	The website content is presented in eight main sections: (1) “Hand and Arm Transplant,” (2) “Process,” (3) “Risks,” (4) “Recovery,” (5) “Options,” (6) “Decision-Making,” (7) “Resources,” and (8) “About Us.”		No change	
	Providing learning guidance	The website uses video testimonials of UE VCA recipients, participants, and candidates as well as UE VCA providers to supplement reading-based learning.		No change	
	Eliciting performance	The “Myths and Facts” section elicits performance by testing users’ knowledge of the accuracy of myth statements.		No change	
	Providing feedback	The “Myths and Facts” section provides feedback by pointing out inaccuracies in the myth statement and providing correct information in the fact statements.		No change	
	Enhancing retention and transfer	The “Question Prompt Sheet” under the “Resources” section provides a list of questions that patients can ask their physicians to learn about hand or arm transplantation. This list of questions enhances the patients’ retention of important UE VCA topics and offers transfer through real-world application in discussion with providers.		No change	
**Website tone and values**					
	Target audience	Participants expressed difficultly identifying the target audience of the website when visiting the home page.		The home page includes a statement that the website is designed to help individuals with UE amputations, their families, and health care professionals make informed treatment decisions.	
**Sitemap**					
	Reading level	Participants expressed difficulty in understanding subsection headers such as “voluntariness” and “psychosocial.”		Sitemap headers that were difficult to understand were replaced with lower-reading–grade-level language such as “optional treatment” and “emotional and social.”	
**Terminology**					
	Language sensitivity	The research team was concerned that the word “amputee” might be offensive to website users as it does not use “people-first” language.		All instances of the word “amputee” on the website were changed to “people with upper limb amputations.”	
	Plain language	Participants urged that the website refrain from using medical jargon.		Website language was changed to use plain language.	
**Images and videos**					
	Diversity	Participants requested that images on the website display UE VCA recipients of diverse sexes, races or ethnicities, and nationalities.		The website included images of recipients from diverse backgrounds, including nationality, age, sex, and race or ethnicity.	
	Cosmetic outcomes	Participants reported wanting to see images of the transplanted limb, including scarring.		Images displaying recipients using their UE VCA to do functional tasks while the scarring of the limbs was visible were included on the website.	
	Functional outcomes	Participants wanted images to showcase UE VCA recipients doing functional tasks that might be difficult to perform living with an amputation.		More action shots were included to show recipients performing activities such as brushing their hair, doing hand therapy, and playing the guitar.	
**Data tables and graphics**					
	Diversity	Participants did not want the website to have the timeline of number of UE VCA surgeries performed in the United States stratified by the transplant recipients’ race and sex.		The graph showing number of UE VCAs performed stratified by recipient race was removed. The graph stratified by sex was retained to assure website users that UE VCA is available to both sexes.	
	Relevance	Participants expressed that graphs showing willingness to authorize deceased donation of one’s own UE or of one’s deceased family member’s UE was irrelevant to them.		The graph of deceased donation willingness rates was removed.	
	Detail	Participants expressed that the table outlining the costs of the UE VCA surgery and medications contained too much information.		Data about the projected cost of taking an immunosuppressive drug regimen were removed from the table.	

^a^UE: upper extremity.

^b^VCA: vascularized composite allotransplantation.

#### Website Design

The website design and content were guided by the instructional design strategies of the conditions of learning by Gagne et al [41]: (1) gaining attention, (2) informing the learner of the objective, (3) stimulating recall of previous learning, (4) presenting the stimulus or content, (5) providing learning guidance, (6) eliciting performance, (7) providing feedback, and (8) enhancing retention and transfer (Table 2). The assessing performance design strategy was not relevant to *Within Reach* as the website does not promote VCA as a treatment option but rather aims to provide users with neutral information for informed decision-making (see Multimedia Appendices 3-7 for screenshots of examples of these strategies).

**Figure 2:**
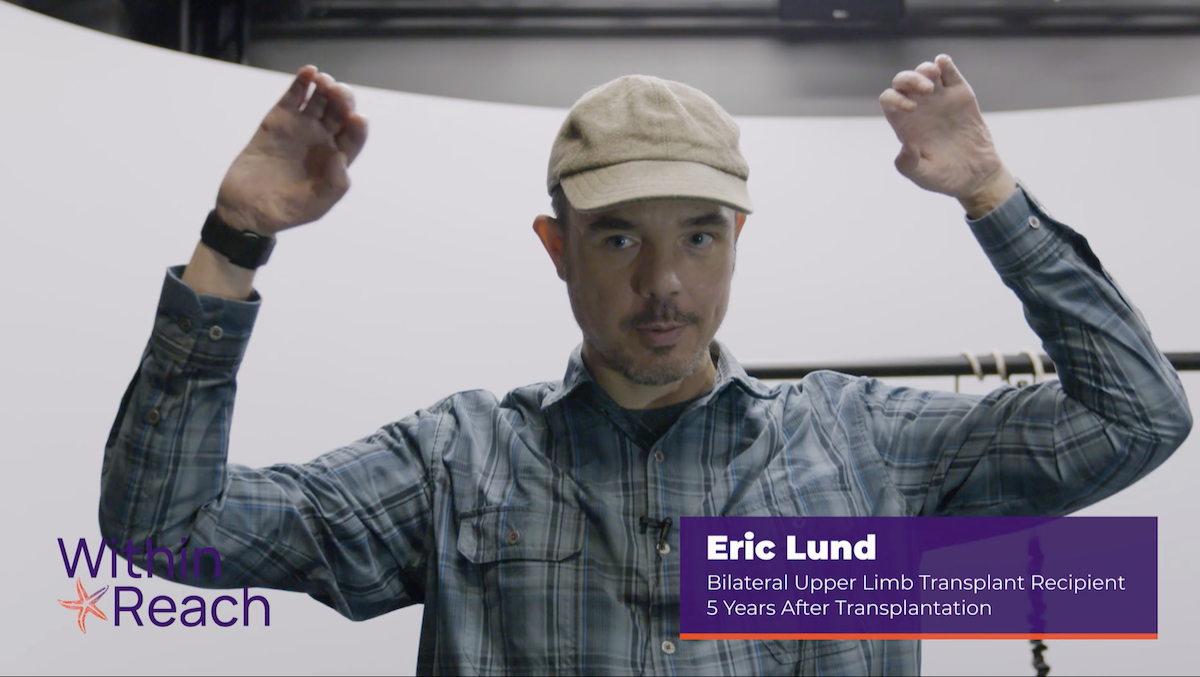
This screenshot illustrates a bilateral upper extremity vascularized composite allotransplantation recipient demonstrating his range of motion.

**Figure 3:**
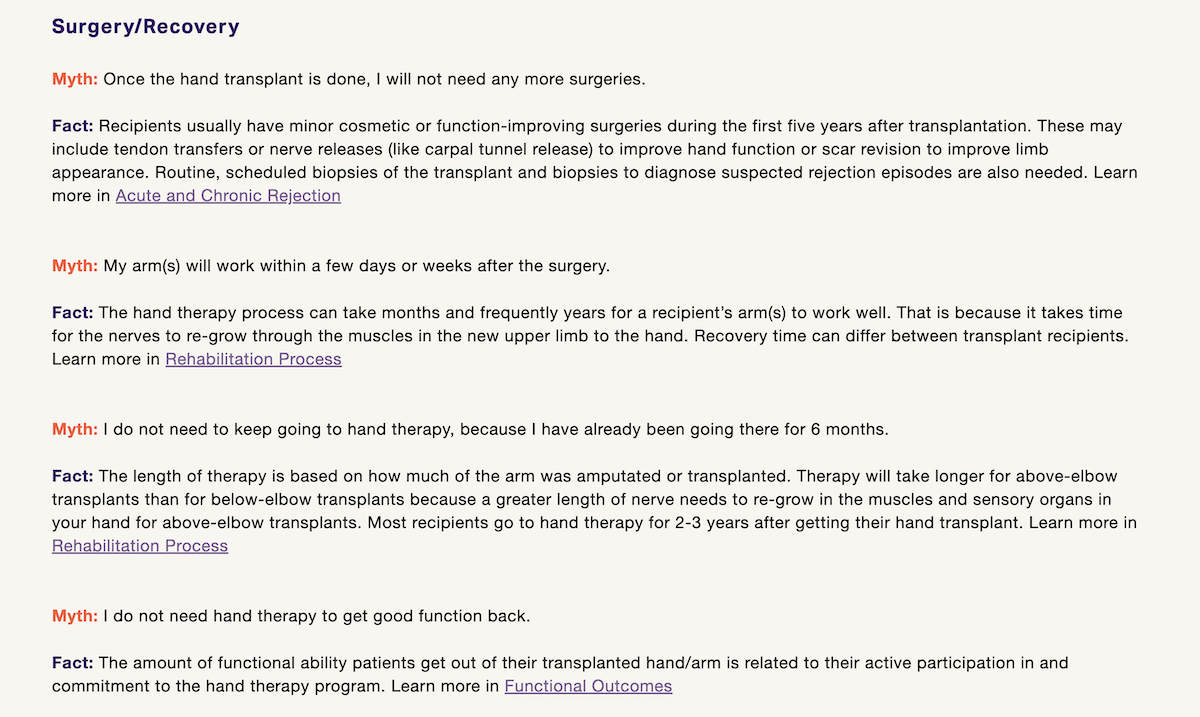
This screenshot illustrates the surgery and recovery section of the Myths and Facts page.

The website gains users’ attention by using thought-provoking questions (ie, “Did you know that the first UE VCA was performed in 1999?”) and attention-grabbing photos or videos on the home page (Figure 2). The website informs users of the objective by incorporating it into the introductory text of each section. The website stimulates recall of previous learning by linking the information between sections. The website presents content about UE VCA as a treatment option and provides learning guidance through opportunities to process the information. The website uses video testimonials of UE VCA recipients, participants, and candidates as case studies for real-world application. The website elicits performance within a *Myths and Facts* section by linking participants to other relevant topic areas. The *Myths and Facts* section provides users with an opportunity to both apply their knowledge of VCA as a treatment option and receive feedback as they separate myths from facts (Figure 3). The *Resources* section provides a question prompt sheet, which is a structured list of questions about UE VCA that can be used by patients when communicating with their physicians [38]. The structured list of questions enhances users’ retention of information about UE VCA and transfers concepts to real-world applications with physician-patient conversations.

#### Website Development

We followed the International Patient Decision Aid Standards version 4.0 (IPDAS v4.0) guidelines for developing the *Within Reach* website. The IPDAS v4.0 groups 44 items into 3 categories of criteria: *qualifying* (6 items), *certifying* (10 items), and *quality* (28 items) [55]. Patient materials are required to meet the 6 *qualifying* criteria to be considered as patient decision aids. The 10 c*ertifying* criteria are considered essential to avoid the risk of harmful bias and are required to meet the certification standards. The remaining 28 items of the *quality* criteria enhance the decision aid but are not required. In total, 2 members of the research staff (KBV and MD) independently evaluated the website using the IPDAS v4.0 criteria. Discrepancies were resolved through discussion. The website met all 6 IPDAS criteria to *qualify* as a patient decision aid and 50% (5/10) of the *certifying* criteria. In total, the 80% (4/5) of *certifying* criteria that were not met were not relevant to our patient decision aid as our website is not a diagnostic test. The remaining unmet criterion required providing an update policy, which was not feasible to meet because of limited grant funding. The website met 43% (12/28) of the *quality* criteria.

The IPDAS criteria are pertinent to ensure that patients are well informed about their treatment options. Although the IPDAS emphasize patient participation in shared decision-making, the use of the *Within Reach* website is expected to be much broader depending on the user. For example, health care providers may draw upon the website to learn facts to share with patients during clinical visits or recommend the site to patients for supplementary information; patients may rely on the website to help them determine whether to pursue UE VCA and aid in finding a transplant program to initiate evaluation. Patients may also refer to the website during their lengthy VCA evaluation process to gather further information about the topics raised during this process.

#### Website Tone and Values

*Within Reach* conveys a neutral position on UE VCA (Table 2). Focus group participants commented on the “neutral” stance of the website, specifically regarding the mission statement. Participants found the website to be “informative” and appreciated that the website content was informed by the input of clinicians and individuals with UE amputations:

I think it’s good that you’re talking about who you spoke to get this information, the purpose so that you’re not trying to say that you’re for or against it. It’s just simply for information and you’ve come from all these different diverse backgrounds to give them the most comprehensive information that you can.

Site 2, focus group 1

The website was designed to be a patient-centered resource that provides evidence-based information about UE VCA and does not attempt to “convince the reader” or “push” individuals toward pursuing VCA. Furthermore, the website does not focus heavily on the potential benefits associated with the procedure and thoroughly covers the risks associated with VCA. A participant discussed the importance of the website including the risks and negative experiences or testimonials of UE VCA recipients “because that would tell me I’m not being sold something. I’m being given a fair piece of information” (site 1, focus group 3). The website includes testimonials by UE VCA recipients recounting their experiences with the recovery process.

Focus group participants appreciated that the website did not exclusively feature information on UE VCA. For example, the website’s *Options* section provides information about alternative options for individuals with UE amputations, including different types of prostheses. Participants reported that the presence of the *Options* section was important on the website, demonstrated neutrality, and made the website feel more trustworthy to individuals with UE amputations.

Several (9/11, 82%) participants at 1 site explicitly stated that they found it challenging to identify the target audience of the website:

It’s a lot of information and as I look through the website it looks like some of it is geared towards amputees and some of the website is geared, it looks like towards medical professionals.

Site 3, focus group 2

Few (3/11, 27%) participants reported that they thought that the target audience for the website was military service members because of the affiliation with WR. Furthermore, few (3/11, 27%) participants found it difficult to determine the purpose of the website:

You really have to read into it [website] to understand what the website is about.

Site 3, focus group 2

...it’s a little confusing trying to figure out what it’s about, until you get to the Purpose section. Without reading, you can’t decipher what’s going on.

Site 3, focus group 2

**Figure 4:**
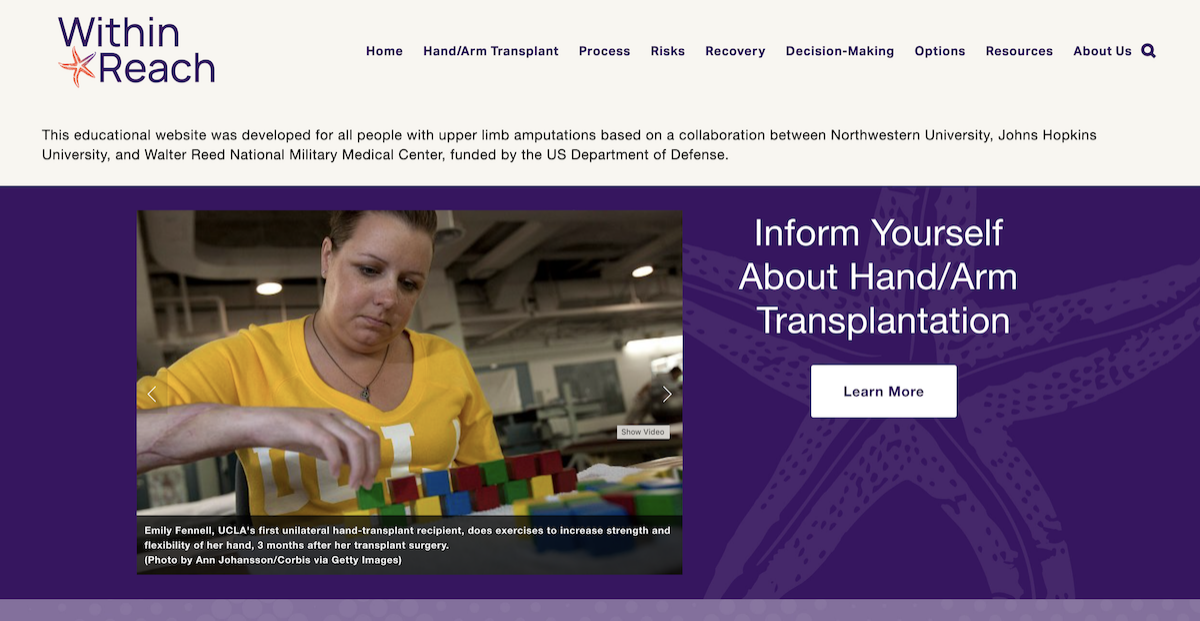
This screenshot presents the top portion of the home page of the Within Reach website. UCLA: University of California, Los Angeles.

On the basis of focus group feedback, the website was revised to include a statement at the top of the home page that explicitly identifies the target audience and the 3 organizations that collaborated to develop the website:

This educational website was developed for all people with upper limb amputations based on a collaboration between Northwestern University, Johns Hopkins University, and Walter Reed National Military Medical Center, funded by the US Department of Defense. (Figure 4)

#### Sitemap

Participants had difficulty understanding some section headers initially listed on the sitemap, specifically words such as “voluntariness” and “psychosocial” (Table 2). Participants were concerned that the verbiage used in the sitemap would be challenging and a “turn-off” to individuals with limited health literacy using the website. The focus group feedback prompted sitemap revision to incorporate more commonly used words, including “optional treatment” instead of “voluntariness” and “emotional and social” instead of “psychosocial.”

#### Terminology

The website developers strived to use terminology that did not stigmatize individuals living with UE amputations (Table 2). *Within Reach* uses the phrase “individuals living with amputations” instead of “amputee.” Being referred to as an “amputee” may be offensive to some individuals living with UE amputations [56]. The website uses people-first language, which recognizes an individual before their diagnosis understanding that individuals are more than a medical diagnosis.

Several focus groups raised concerns about the medical terminology used on the website:

Keep it simple and keep the doctor’s terminology out of there.

Site 3, focus group 1

Focus groups requested that we use “plain English” (site 1, focus group 3) and that we “lower the complexity of the words” (site 1, focus group 2). The focus group prompted revisions to the website to incorporate plain language where appropriate, including not using the term *vascularized composite allotransplantation*.

#### Images and Videos

*Within Reach* features photographs of individuals from varied racial, ethnic, and gender backgrounds (Table 2). Focus groups consistently requested that the website display images featuring individuals from diverse backgrounds, particularly on the home page. The research team intentionally searched for photos of transplant recipients and intentionally selected individuals with UE amputations of diverse backgrounds to be videotaped for representation on the website. Participants indicated a preference for photographs that displayed the “realness” and “scarring” associated with VCA:

I don’t want a false sense of reality. I like how it shows the raw hands, the scars. You know, versus people expecting it to be basically, like a miracle where you don’t have any scars, you’re not going to—you know what I mean? So, I like the reality of it.

Site 2, focus group 3

Participants discussed the importance of photographs reflecting “both sides of [the transplant]” experience and not focusing solely on the positive or negative aspects of the experience. Photographs with scarring that are “in your face” may prompt some individuals with UE amputations to reconsider whether a VCA transplant is the right choice for them. Participants reported that the website should display photographs of recipients using their transplanted arm to perform recreational activities and activities of daily living, which have been incorporated into the website. The website features 29 photographs of VCA recipients, prostheses, and prosthesis users.

The website includes video testimonials from clinicians; individuals with UE amputations; and VCA candidates, participants, and recipients. The testimonials include a VCA recipient discussing experiences with rejection and the challenges of being a VCA recipient. The website hosts 108 patient videos including 2 bilateral recipients and 80 health care provider videos (occupational therapists, transplant social workers, hand surgeons, and hand or arm transplant surgeons). See Multimedia Appendix 8 for screenshots of patient and health care provider videos.

#### Data Tables and Graphics

Focus groups consistently preferred tables that displayed data on the age of VCA recipients, were less enthusiastic about reporting VCA recipients by gender, and disliked tables on recipients by race owing in part to the incomplete race or ethnicity data reported by the Organ Procurement and Transplant Network or United Network for Organ Sharing (Table 2). Participants perceived information on the age of recipients as useful to individuals with UE amputations weighing the decision to pursue UE VCA at their age:

If other people did it at my age like this, and they’re successful, then that could determine my decision on it.

Site 1, focus group 3

Some focus groups expressed difficulty understanding how data on recipients by gender were relevant and important to people’s decision-making process. Participants suggested that tables on gender and race should not be included on the website. We retained the gender table to ensure that future viewers could recognize that all genders are eligible to receive a UE VCA.

**Figure 5:**
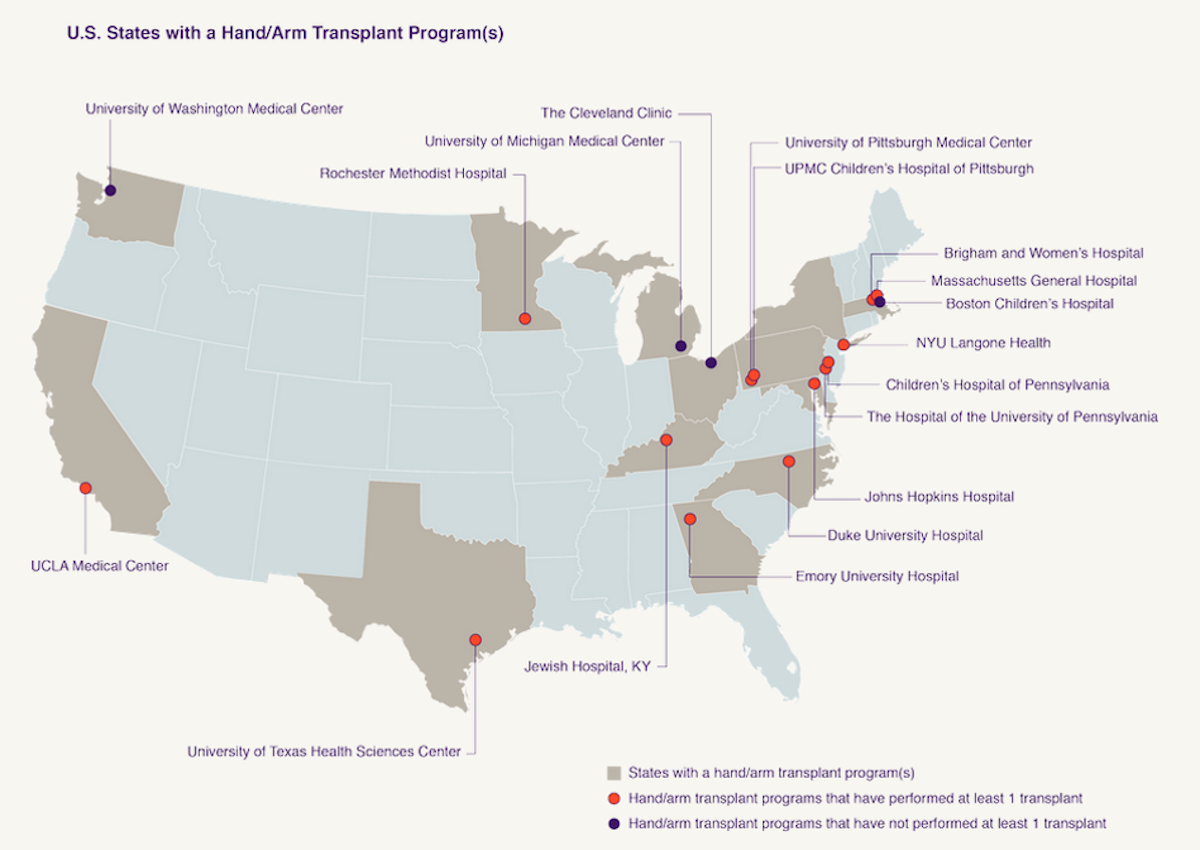
This screenshot illustrates a resource that enables users to identify hand and arm transplant programs in the United States. KY: Kentucky; NYU: New York University; UCLA: University of California, Los Angeles.

Most focus groups preferred the graphics displaying the location of transplant centers in the United States with established UE VCA transplant programs, including those programs that have not yet performed a transplant (Figure 5). Participants also appreciated the information on the number of UE transplants performed in the United States by transplant center. Participants across focus groups reported that such information would assist individuals considering UE VCA in identifying programs near their geographic location, specifically those with the most experience performing bilateral and unilateral transplants.

Several focus groups were surprised by a table displaying the number of individuals with UE amputations on the waiting list for UE transplants in the United States and the number of UE recipients in the United States and worldwide. Focus groups assumed that the numbers presented in the tables were incorrect—“this graph can’t be right” (site 1, focus group 3)—and participants expected the number of recipients and those on the waitlist to be higher:

Yeah, I would’ve thought that those numbers would be higher; that there would be more of these procedures performed.

Site 1, focus group 3

Focus group participants expressed concern that so few people had received a UE transplant, that it is not a common procedure, and concluded that the procedure would be risky:

When I think of it, there’s a lot of unknowns for me as to the potential risks of having it done.

Site 2, focus group 1

Focus groups noted that the limited number of UE recipients would be worth bearing in mind when considering UE VCA as a treatment option “because not many have done it, I would not be comfortable doing it” (site 2, focus group 3) and “for me to decide if I ever have a transplant, I would want to know if other people have had it” (site 3, focus group 3). Some (7/26, 27%) participants reported that they perceived the low number of individuals on the waiting list in the United States to mean that they would not have to wait long for the surgery.

Focus groups consistently did not prefer graphs displaying information about willingness to authorize deceased donation of one’s own UE or of one’s deceased family member’s UE as that information was not considered “worthwhile” or relevant to decision-making about receiving a UE VCA. Accordingly, tables on deceased donation were not included. Several focus groups found the table on the cost of UE VCA to be “intimidating” and suggested the “need to dummy it down with a breakdown of the cost, make it simple” (site 3, focus group 1). The revised cost table was simplified by removing the detailed immunosuppressive drug regimen.

### Usability Testing

#### Demographics

Of the 24 eligible participants contacted, 14 (58% participation rate) completed usability testing. Those completing usability testing were evenly divided across NU and WR. Usability testing was conducted until saturation was reached. In total, 4% (1/24) of the participants, who were contacted and scheduled for an interview, were not interviewed once saturation was reached.

Participants had a mean age of 50 years, and most were male (11/14, 79%), White (9/14, 64%), and literate (13/14, 93%) and had undergone a unilateral amputation (12/14, 86%; Table 3). The mean number of years since the first amputation was 18 (SD 17.3; range <1-53) years. There were 7% (3/44) of WR participants and 9% (4/44) of NU participants who took part in both a focus group and usability testing.

**Table 3:** Usability testing participants’ sociodemographic characteristics (n=14).

Characteristics			Total		NU^a^ (n=7)		WR^b^ (n=7)
Age (years), mean (SD; range)			50.0 (12.0; 27-66)		56.6 (6.7; 45-66)		42.7 (12.4; 27-65)
**Sex, n (%)**							
	Male	11 (79)		6 (86)		5 (71)	
	Female	3 (21)		1 (14)		2 (29)	
**Ethnicity, n (%)**							
	Not Hispanic or Latino	13 (93)		7 (100)		6 (86)	
	Hispanic or Latino	1 (7)		0 (0)		1 (14)	
**Race, n (%)**							
	White	9 (64)		5 (71)		4 (57)	
	Black or African American	2 (14)		1 (14)		1 (14)	
	Native Hawaiian or other Pacific Islander	1 (7)		1 (14)		0 (0)	
	Multiracial	2 (14)		0 (0)		2 (29)	
**Marital status, n (%)**							
	Married or domestic partner or civil union	12 (86)		6 (86)		6 (86)	
	Separated or divorced	1 (7)		0 (0)		1 (14)	
	Never married or single	1 (7)		1 (14)		0 (0)	
**Education, n (%)**							
	Some college	5 (36)		2 (29)		3 (43)	
	College graduate	5 (36)		2 (29)		3 (43)	
	Postgraduate degree	4 (29)		3 (43)		1 (14)	
Health literacy (adequate), n (%)			13 (93)		7 (100)		6 (86)
**Employment status, n (%)**							
	Employed full time	7 (50)		3 (43)		4 (57)	
	Employed part time	1 (7)		1 (14)		0 (0)	
	Homemaker	1 (7)		0 (0)		1 (14)	
	Disabled	1 (7)		1 (14)		0 (0)	
	Retired	4 (29)		2 (29)		2 (29)	
**Income (US $), n (%)**							
	<15,000	0 (0)		0 (0)		0 (0)	
	Between 15,000 and 34,999	1 (7)		1 (14)		0 (0)	
	Between 55,000 and 74,999	1 (7)		1 (14)		0 (0)	
	Between 75,000 and 94,999	5 (36)		1 (14)		4 (57)	
	>95,000	5 (36)		3 (43)		2 (29)	
	Prefer not to answer	2 (14)		1 (14)		1 (14)	
**Primary health insurance, n (%)**							
	Private	4 (29)		3 (43)		1 (14)	
	Tricare	5 (36)		0 (0)		5 (71)	
	Medicaid or Medicare	4 (29)		3 (43)		1 (14)	
	None	1 (7)		1 (14)		0 (0)	
**Health status, n (%)**							
	Excellent	5 (36)		3 (43)		2 (29)	
	Very good	5 (36)		1 (14)		4 (57)	
	Good	3 (21)		3 (43)		0 (0)	
	Fair	1 (7)		0 (0)		1 (14)	
**Dominant hand amputated, n (%)**							
	Yes	12 (86)		7 (100)		5 (71)	
	No	2 (14)		0 (0)		2 (29)	
**Upper extremity amputated, n (%)**							
	Right	7 (50)		3 (43)		4 (57)	
	Left	5 (36)		2 (29)		3 (43)	
	Both	2 (14)		2 (29)		0 (0)	
**Amputation type, n (%)**							
	Unilateral	12 (86)		5 (71)		7 (100)	
	Bilateral	2 (14)		2 (29)		0 (0)	
**Amputation level, n (%)**							
	Below elbow	7 (50)		5 (71)		2 (29)	
	Above elbow	6 (43)		1 (14)		5 (71)	
	Below elbow and above elbow	1 (7)		1 (7)		0 (0)	
**Current prosthesis type^c^, n (%)**							
	Mechanic	8 (57)		3 (43)		5 (71)	
	Myoelectric	6 (43)		4 (57)		2 (29)	
	None	1 (7)		0 (0)		1 (14)	
**Time since the last amputation (years), n (%)**							
	<2	2 (14)		1 (14)		1 (14)	
	2-8	2 (14)		0 (0)		2 (29)	
	9-15	2 (14)		1 (14)		1 (14)	
	16-25	4 (29)		2 (29)		2 (29)	
	>25	4 (29)		3 (43)		1 (14)	

^a^NU: Northwestern University.

^b^WR: Walter Reed National Military Medical Center.

^c^Percentages do not add up to 100 as some participants used multiple prostheses.

#### Impressions

Participants reported an overall positive impression of *Within Reach*, conveyed in terms of it being “informative,” “organized,” and “interactive.” In addition, most found the website easy to navigate:

...it seems to be really pretty straightforward just when you open the page and like you can find what you want at the top. So, like you have your drop-down, you can click on what you want to click on...Pretty easy to navigate.

Site 3, WR 036

A participant with a bilateral amputation said the following regarding their ability to navigate the website:

I found it easy to navigate, especially using a mouse or trackball.

Site 1, NU 021

Participants consistently reported that the website was sensitive to people with UE amputation:

I like the feel of this website. I think it addresses the needs of people with limb loss and limb difference, a neutral way.

Site 1, NU 019

It seems like there was a pretty good understanding of experiences that folks have as far as how you view yourself and limb loss and stuff like that, so that seems pretty insightful and straightforward. So, I appreciated that.

Site 3, WR 020

Participants found the website to be “neutral” or “unbiased” regarding the portrayal of UE transplantation as a treatment option for people with UE amputations. A participant stated the following:

So internally what I was doing was I was looking at the different sections that you had and was there a balance between pros and cons and availabilities and looking at this seems like very, you know, very neutral. And again, my nuances are definitely in the weeds based upon my prior experience history of being an amputee and involvement with the different communities and medical communities and things of that nature.

Site 1, NU 021

Most participants’ favorite section of the website was the *Options* section, suggesting that people living with a UE amputation had more to learn about their alternative treatment options. A participant noted the following:

The Options page was very interesting and [I] keep coming back to that because then it better informs me as to why my doctor’s notes are decisions and offered up certain options that I was, you know, given, like I was not given the battery-powered [prosthesis] option that was never made available to me.

Site 1, NU 039

Overall, participants reported that they found the website culturally sensitive to the target population of people with UE amputations (Table 4). Participants rated their overall experience highly, with a mean score of 6.1 (SD 0.91; range 4.0-7.0). Few (3/14, 21%) participants provided lower ratings on overall experience as they were not personally considering UE transplantation as a treatment option and, hence, would not be using the website or they did not recall seeing images of African American patients on the site. Participants expressed satisfaction with the website content featured and did not perceive that information was missing or should be removed:

I think you got everything; the tutorials are one of the best. I always think that tutorials are one of the best things to use because you can go with not looking at it, but you listen to people.

Site 3, WR 011

**Table 4:** Cultural sensitivity (n=14).

Question	Strongly agree, n (%)	Agree, n (%)	Neutral, (%)	Disagree, n (%)	Strongly disagree, n (%)
The words, phrases, and expressions are familiar to the intended audience.	8 (57)	5 (36)	0 (0)	1 (7)	0 (0)
The words, phrases, and expressions are free from stereotypical meaning.	8 (57)	5 (36)	1 (7)	0 (0)	0 (0)
The message is linked to sources credible to the intended audience.	8 (57)	2 (14)	2 (14)	1 (7)	1 (7)
The message addresses stereotypes and myths.	8 (57)	4 (29)	2 (14)	0 (0)	0 (0)
The graphics accurately depict the physical features (eg, hairstyle and clothes) of the intended audience.	6 (43)	4 (29)	3 (21)	1 (7)	0 (0)
Symbols are representative of the intended audience.	3 (21)	7 (50)	4 (29)	0 (0)	0 (0)
The stature and poise of the individual is representative of the gender and social roles of the intended audience.	4 (29)	8 (57)	1 (7)	1 (7)	0 (0)
The educational materials are culturally sensitive to ethnic minority communities.	3 (21)	4 (29)	5 (36)	2 (14)	0 (0)
The website materials seemed neutral and unbiased; that is, the website was neither in favor of nor against hand or upper limb transplantation.	8 (57)	4 (29)	0 (0)	2 (14)	0 (0)

#### ASQ Results

The ASQ mean scores for each task scenario ranged from 1.3 to 2.3 (Table 5). The mean findability scores for each task ranged from 3.4 to 4.7. The mean satisfaction scores for each task ranged from 3.6 to 4.4.

**Table 5:** Satisfaction scores of usability testing participants (n=14).

		Total, mean (SD; range)	NU^a^ (n=7), mean (SD; range)	WR^b^ (n=7), mean (SD; range)
**ASQ^c^**				
	Task scenario 1^d^	2.0 (1.5; 1.0-7.0)	1.9 (0.6; 1.0-2.7)	2.0 (2.2; 1.0-7.0)
	Task scenario 2^e^	1.3 (0.6; 1.0-3.0)	1.5 (0.7; 1.0-3.0)	1.1 (0.4; 1.0-2.0)
	Task scenario 3^f^	2.0 (1.2; 1.0-4.3)	2.4 (1.0; 1.0-4.0)	1.6 (1.2; 1.0-4.3)
	Task scenario 4^g^	2.3 (1.2; 1.0-4.7)	2.4 (1.4; 1.0-4.7)	2.2 (1.1; 1.0-4.0)
	Task scenario 5^h^	1.4 (0.8; 1.0-4.0)	1.1 (0.2; 1.0-1.3)	1.6 (1.1; 1.0-4.0)
SUS^i^		88.9 (10.6; 70-100)	87.5 (11.1; 70-100)	90.0 (10.7; 75-100)
NPS^j^		9.6 (0.6; 8-10)	9.6 (0.8; 8-10)	9.7 (0.5; 9-10)
Overall website experience^k^		6.1 (0.9; 4.0-7.0)	6.1 (0.7; 5.0-7.0)	6.0 (1.2; 4.0-7.0)

^a^NU: Northwestern University.

^b^WR: Walter Reed National Military Medical Center.

^c^ASQ: After-Scenario Questionnaire.

^d^Task 1: What functions and sensations can you expect to gain after getting a hand or arm transplant?

^e^Task 2: How can you tell if you are qualified to get a hand or arm transplant?

^f^Task 3: There are a lot of pros and cons to a hand or arm transplant, and people have to figure out whether this is the right option for them. How do you know if this option is worthwhile for you?

^g^Task 4: Hand or arm transplant recipients might experience changes in mood after the transplant. What are different ways that recipients emotionally respond to the transplant?

^h^Task 5: What does hand therapy involve and for how long do recipients need to do it?

^i^SUS: System Usability Scale.

^j^NPS: Net Promoter Score.

^k^“Rate your overall experience with this website” assessed overall website experience on a scale of 1 to 7.

#### SUS Results

The mean SUS score was 88.9 (SD 10.6; range 70-100; Table 5). All (14/14, 100%) participants recorded an above-average SUS score (>68).

#### NPS Results

Participants reported an overall NPS score of +93 (Table 5). Most (13/14, 93%) scores were classified as “promoters,” with a rating of 9 or 10, and the score of 7% (1/14) of the participants was classified as “passive,” with a rating of 8. The mean NPS score was 9.6, indicating a high likelihood that participants would recommend the website based on their experience.

#### Suggested Changes and Website Revisions

Participants recommended changing website content, format, and functionality by adding photographs of an individual of African ancestry to the carousel on the home page, adding graphic photos, adding disclaimers to the *Patient Experience* pages, inserting more space in the *Myths and Facts* section, inserting hyperlinks to connect website sections to each other, adding a voice-over capacity, and improving the search function capability. We were able to accommodate all the recommendations except for 3. Specifically, we did not provide voice-over capacity throughout the website as individual computer settings can adjust for this. A participant stated the following:

I mean really if you’re going to add anything, maybe a voice command type of thing. But not as detailed as like a Siri, but something more focused with keywords or even like in your glossary of terms. I think it would–wouldn’t be as frustrating if somebody didn’t have the dexterity.

Site 3, WR 035

In addition, we did not include graphic photos on the website as participants had conflicting views on whether to include graphic photos or images of the UE VCA procedure. Participants recommended a chart comparing the costs of prosthetic options, but we did not make this change as costs are affected by insurance coverage and individual patient circumstances. On the basis of feedback, we changed the format of the video drop-downs at the bottom of each page from a horizontal bar to underlined text with a left-justified header to make the drop-down feature more apparent. In addition, pages with larger paragraphs were broken down into several easier-to-digest bullets.

## Discussion

### Principal Findings

The *Within Reach* website was successfully developed with the input of UE VCA stakeholders and civilians and service members with UE amputations. Our educational website aims to inform individuals with UE amputations, their families, and health care providers about UE VCA as a treatment option. This manuscript outlines the process undertaken to design and develop *Within Reach.* Our findings demonstrate that the *Within Reach* website prototype was easy to navigate and constituted an unbiased resource for individuals with UE amputations, who reported favorable experiences and high user satisfaction.

### Comparison With Prior Work

*Within Reach*’s SUS score of 88.9 compared highly with the widely accepted mean SUS score of 68 [57], indicating that *Within Reach* is a well-designed, functional application with high usability and satisfaction among people with UE amputations. *Within Reach* scored similarly to other health-oriented websites and applications [58,59]. Previous research indicates that a score >68 would be considered above average; a score >80.3 represents the top 10% of scores and would be considered an excellent score, whereby individuals are most likely to recommend the website [60].

The NPS score of +93 reflects high acceptance and satisfaction with *Within Reach* among study participants. Although an NPS score of 50 is considered excellent, our NPS score indicates that satisfaction with the website was very high and reflects a high likelihood that participants will recommend *Within Reach* to friends and family.

### Clinical Applications

We recommend integrating *Within Reach* into clinical practice as a supplement to patient-provider education. Health care professionals, including hand or upper limb surgeons, prosthetists, hand therapists, and UE VCA teams, should inform individuals with UE amputations about the website as a useful educational resource before scheduled amputations or during the rehabilitation period after traumatic (unexpected or accidental) amputations. Health care professionals could download the brochure about *Within Reach* from the website and distribute it in the clinic setting. By reviewing *Within Reach* before clinic visits, individuals with UE amputations can reflect on their values and beliefs to guide their treatment preferences and thereby be well poised to engage in more informed discussions and shared decision-making with their health care professionals multiple times over the course of their lengthy rehabilitation process while recovering from their amputation.

We also recommend that health care professionals rely on *Within Reach* as an educational resource for their own edification. Owing to the small number of UE VCA recipients in the United States, it is likely that health care professionals do not have sufficient knowledge of UE VCA to educate patients about it. Thus, health care professionals may use *Within Reach* to gain foundational knowledge for informing individuals with UE amputations about their treatment options.

### Lessons Learned

We learned many lessons in developing the *Within Reach* website in collaboration with the upper limb loss community. Our participants with UE loss conveyed how important it is to recognize UE VCA recipients as credible and valued sources of information. The recipients’ experiences, positive and negative, should be promoted throughout VCA education. We recommend developing digital resources for the UE loss community as participants expressed challenges in handling paper owing to their UE loss or prosthetic use. In addition, we recommend that UE VCA materials be presented neutrally, highlighting information and images that demonstrate the process.

We also realized that recruiting participants with UE loss is exceptionally challenging. The UE loss community is small. In addition, this is a heavily studied population as many initiatives aim to help improve the lives of those with limb loss. Therefore, some health care providers and support group communities were not receptive to disseminating information for study participation among the qualified individuals within their community. Moreover, snowball sampling was not an effective recruitment strategy for this population.

### Future Research

Future research should evaluate whether the use of *Within Reach* can effectively increase knowledge of UE VCA and enhance informed treatment decision-making regarding UE VCA among individuals with UE amputations. In total, 2 study leaders plan on establishing a panel of VCA experts to review the website annually to recommend updates to the website content. The website developer has trained the research team to edit the website and implement changes as needed.

### Strengths

The strengths of this study include the multisite design and participant recruitment and representation from geographically diverse US regions, suggesting the generalizability and transferability of the findings. The mixed methods design enabled us to qualitatively and quantitatively assess perceptions of the *Within Reach* website. The study engaged a large number of individuals with UE amputations and UE VCA candidates, participants, and recipients to inform website content and navigability so as to ensure a patient-centered website. In addition, the website is accessible for people with disabilities and follows the guidelines of the Health On the Net Foundation certification [36].

### Limitations

This study has several limitations. The study sample comprised primarily White, middle-aged, well-educated men several years after amputation, which may limit the transferability of our findings to individuals with UE amputations of different racial, ethnic, and gender backgrounds. Usability testing was conducted in person and using a videoconferencing platform; remote testing may have made observation of participants’ navigation more challenging in terms of detecting subtle nonverbal communication in body posture and facial expression. Our data suggest that SUS ratings differed slightly between UE VCA candidates, participants, and recipients compared with individuals not interested in pursuing VCA. A larger study sample of UE VCA participants and candidates would afford greater power to assess this. The website content was written above the sixth grade reading level given the subject matter terminology and complexity, which may make it difficult to comprehend [61]. Participation included only English speakers; the website may not reflect the views of non–English-speaking individuals with UE amputations. As our sample included mostly individuals who were not interested in UE VCA, it is unlikely to have been affected by selection bias.

### Conclusions

*Within Reach* is a patient-centered educational resource about UE VCA targeted to people with UE amputations, their families, and health care professionals to support informed treatment decision-making. Health care providers can inform individuals recovering from UE loss about *Within Reach*. A brochure about the website can be downloaded and distributed to people with UE amputations. Future research should assess whether *Within Reach* improves knowledge about UE VCA and enhances informed decision-making about UE VCA as a treatment option.

## Appendix

Multimedia Appendix 1 Focus group moderator guide.

Multimedia Appendix 2 Website materials presented during the focus groups and associated questions about the website.

Multimedia Appendix 3 Screenshot of one of the many videos of a hand and arm transplant surgeon.

Multimedia Appendix 4 Screenshot of an upper extremity vascularized composite allotransplantation candidate explaining why he decided to pursue a hand and arm transplant.

Multimedia Appendix 5 Screenshot illustrating various Did you know? questions used to engage website users. Different questions appear in different website sections.

Multimedia Appendix 6 Screenshot showcasing the experiences of upper extremity vascularized composite allotransplantation recipients by presenting quotes from different recipients in various sections throughout the website.

Multimedia Appendix 7 Screenshot illustrating the topic areas featured under the risks of hand and arm transplantation section.

Multimedia Appendix 8 Health care professionals and an upper extremity vascularized composite allotransplantation recipient discussing the qualities of a good candidate for hand and arm transplantation.

## Abbreviations

ASQ: After-Scenario Questionnaire
IPDAS v4.0: International Patient Decision Aid Standards version 4.0
JHU: Johns Hopkins University
NPS: Net Promoter Score
NU: Northwestern University
SUS: System Usability Scale
UE: upper extremity
VCA: vascularized composite allotransplantation
WR: Walter Reed National Military Medical Center

## Data Availability

The data sets generated and analyzed during this study are not publicly available because of the lack of available funding but are available from the corresponding author upon reasonable request.
