# Supplementary material for: A Patient-Centered Website (Within Reach) to Foster Informed Decision-making About Upper Extremity Vascularized Composite Allotransplantation: Development and Usability Study
Source: JMIR Form Res. 2023 Feb 7;7:e44144. doi: 10.2196/44144 (PMC9944141; doi:10.2196/44144)
Supplement: Multimedia Appendix 1 [file formative_v7i1e44144_app1.docx]

**Supplementary File 1. Telephone Focus Group Moderator’s Guide**

Introduction

Welcome to this group discussion. My name is [name] and I am the moderator. My role is to help start a conversation and to make sure we cover important topics that we would like your input on. I am assisted by _____ and _____.

Focus of Discussion:

Our goal is to hear your thoughts about an educational website we are making about hand and upper limb transplantation. I’d like to first give you some background information about hand transplantation.

As you may know, current treatment options for upper limb amputations include prosthetics, robotics, and reconstructive surgery. Since prosthetics offer limited limb function, sensation, and use, some people with amputations are interested in alternative options.

Vascularized Composite Allotransplantation, also known as ‘VCA,’ are a group of solid organ transplants such as the hand, face, penis, and uterus. Hand and upper limb transplantation is a life-enhancing treatment option for people with upper limb amputations. Hand transplantation can restore hand motor function and sensation. The recipient receives a new hand or arm from a deceased donor – that is, someone who has passed away.

Because hand/upper limb transplantation has been available since 1999, there is no single source of information about it. Also, different healthcare providers have different levels of knowledge and opinions about hand transplantation. As a result, people with upper limb amputations may not be well informed about this option, which can make it hard for them to make a treatment decision.

To help address the need for information about hand transplantation, we are making a website for people with upper limb amputations to learn about hand transplantation, and help them see if hand transplantation is a good choice for them.

Purpose of the Focus Group:

- We are in the [early/middle/end] stages of making the website.
- We are going to talk about your ideas for making the website content and design clearer and sensitive to the needs of people with upper limb amputations.

Before we begin, I want to make clear that:

- You are the experts and we are here to learn from you.
- If you do not understand any question, please let me know and I will rephrase it.
- We’ve mailed you a packet of information and images that we’d like your feedback on. We’ll refer to it later on.
- _____ and _____ are here to assist and will be taking some notes during our discussion.
- If you encounter technical issues, please contact _____.
- As a reminder, we are audio-recording what you say so that we don’t miss anything important.
- We expect our meeting to last up to 2 hours. The last 10 minutes will be reserved for the post-focus group survey.

There are a few “ground rules” we ask your help with keeping:

- I might move you along in conversation. Since we have limited time, I’ll ask that questions or comments off the topic be answered after the focus group session.
- I’d like everyone to speak, so I might ask people who have not spoken to comment.
- Let’s be sure to respect each other and have only one person speak at a time.
- Also, please respect each other’s opinions. There is no right or wrong answer to the questions. It’s okay to have different opinions.
- To keep the discussion confidential, let’s avoid using names or other identifying information.
- I will refer to whatever name you want me to call you – feel free to update your name in Zoom to whatever you would like to be called.
- After the discussion ends, I’ll ask you to fill out a brief post-focus group survey online about your views of the discussion. You can access the online survey with the link that _____ will send to you via email. When you’re done with that, we will compensate you for your time.

Do you have any questions so far?

Okay, now let’s begin with introductions. [5 minutes]

Briefly tell us your name – it can be a pseudonym/fake name, and tell us if you have a unilateral or bilateral amputation, and how long ago it occurred.

**QUESTION GUIDE**

**Awareness [3-5 min] 00:10**

*First, let’s talk about hand transplantation.*

- Has anyone heard about hand transplants before?
- Based on what you’ve heard before or today, how do you feel about hand transplantation?

**Website Name [3 min] 00:13**

*Now, let’s talk about the design of the website draft.*
*Please turn your paper packet to page x, section y.*

- What do you think of this name of the website?

**Website Logo [3 min] 00:16**

*Now, let’s go to page x, section y. Here is the website logo.*

- What do you think about it?

**Mission, Vision, Purpose Statement [6 min] 00:19**

*Now, go to page x, section y.
Take a moment to read through this paragraph.*

- What do you think of this text and how could we improve it?
- What term should we use – Mission, Vision, or Purpose Statement?

**Website Site Map [5 minutes] 00:25**

*Next, go to page x, section y.*

*Read pages x through y.*

*Take a minute to review the content on this slide.*

Here is our site map:

1. What do you think of the topics covered?
2. Are topics in the right order?
3. What topics should we add? What topics should we delete?
4. Can you think of better names for the topics?
5. *If a new topic is recommended, ask:* What information should be covered in each section?

**Terminology Preferences [15 min] 00:30**

*Now, let’s turn to page x, section y. There are a lot of different ways to talk about hand or arm transplantation and we’d like your input on which words or phrases are most user-friendly.*

1. Should we use the acronym “VCA” (Vascularized Composite Allotransplantation) in the website?

Do people need to know about VCA as a category of organ transplants (like hand, face, uterus, and penis transplants)?

[*Show website to group*]

1. What is the best way to refer to Upper Limb VCA?
   1. ‘Hand transplantation’
   2. ‘Upper limb transplantation’
   3. ‘hand/arm transplantation’
   4. ‘your/my new hand
   5. ‘new limb’
2. “Graft’ survival”:
   1. Do people know what ‘graft’ means?
   2. What does ‘graft survival’ mean to you?

      Website text example: “In a study reporting on upper limb transplantation outcomes and survival rates, there were no deaths in the 50 patients who received an upper limb transplant. In these 50 patients, the graft survival rate of their transplanted limbs was 90%.”
3. “Evaluation” – When you hear the phrase “transplant evaluation process,” what does “evaluation” mean to you?
4. When referring to upper limb transplant researchers, which term should we use?
   Which term do you like best?
   1. ‘medical researchers’
   2. ‘scientists’
   3. ‘transplant researchers’
   4. ‘research doctors’
5. When we refer to the medicine that hand transplant recipients take to prevent rejection, what is the best term to use?
   1. ‘Anti-rejection medication’
   2. ‘Anti-rejection medicine’
   3. ‘Immunosuppressants’
6. There are different words to describe the therapy a hand transplant recipient goes through after getting a transplant. One option is ‘hand therapy.’
   1. What does ‘hand therapy’ mean to you?
   2. The term ‘hand therapy’ is often used by providers, but we want to make sure that we use the term that most people will be comfortable with.

Options are:

1. ‘Hand therapy’
2. ‘Physical therapy’
3. ‘Occupational therapy’
4. ‘Rehabilitation’

Which do you like best?

1. Transplant success:

a. When you hear the concept of ‘transplant success,’ what does that mean to you?

b. Here are three ways one could interpret the concept:

1. Surgical success in terms of attaching the hand to the body
2. Hand function gained from the transplant
3. The relevant function gained from a transplant vs a prosthetic

Which interpretation fits your idea of success better?
Do you have other ideas for what success means?
What counts as success?

**Myth/Fact Section [15 min] 00:45**

*Now, go to page x, section y.
Take a moment to read through these slides.*

- What do you think of this section?
- How can we improve it? What needs to be changed?
- Do the answers sound appropriate?
- What other myths should we add?
- Do you like the myth/fact format? Or would you prefer a Frequently Asked Questions format?

**Data Tables & Graphics [12 min] (10 slides) 01:00**

*Now, let’s go to page x, section x.*

*Here are diagrams and tables that present some data.*

*For each table/diagram:*

- Can you please tell us what the table means to you?
- How can we improve the table/graphic?
- What other data tables should we add? What would you like to know?

**Photographs [10 min] 01:12**

*We are going to show you 2 pages of photos.*
*Disclaimer: Some of the photos may be uncomfortable to see.*

- Which photo do you like the best?
- What other pictures or images should we put on the website?
- Do you prefer photographs or technical drawings?

**General Feedback – Website Screenshots [15 min] 01:22**

*Here are some examples of the website – show the ‘Home Page’ and ‘Introduction to VCA’.*

What do you think of the presentation/design?

[*Purple section*] What would you like to see or learn about in this section?

[*Review drop-downs on webpage*] What do you think of how this works?

- What are your impressions of these website drafts (wireframes)? Do they look in logical order?
- How can we make the website look like it is made especially for people with upper limb amputations?
- **How can we make the website more sensitive to the needs of people with upper limb amputations – in terms of using the website, and in the content and design.**
- How can we make the website more sensitive to the needs of people of diverse ethnic and racial backgrounds?
- How could we make the language sound more empowering to people with upper limb amputations?
- What would help encourage people with upper limb amputations to go to the website?
- **What makes a website easy for you to navigate? What features does it have?**

Comprehensibility

- What phrases are hard to understand?
- How can we make it easier to understand?
- Is there anything we can get rid of?

**VCA Recipient Quotes [8 min] 01:37**

*Now turn to page x, section y.*
*Here are 8 quotes from VCA recipients. Please take a moment to read through them.*

- Which quotes do you like best?
- Are there any quotes we should cut?

**Conclusion [5 min] 01:45**

*We’ve got to wrap up our discussion in the next 5 minutes.*

I’d like to use this time to ask each of you two general questions:

- Can you give me a final statement summarizing your recommendations for information and topics to go into the website?
- What do you think the most important elements of the discussion have been?

**Online Survey [5-10 min] 01:50**

Now, _____ will email you a link to the brief post-focus group survey.

Before we hang up, can you please confirm that you received the survey?

Please take a few minutes to complete the survey. It will take 5-10 minutes to complete.

After completing the survey, you will receive a $$ e-gift card.

This concludes the focus group. Thank you very much for your participation. Your input has been most helpful.
