# Supplementary material for: A Patient-Centered Website (Within Reach) to Foster Informed Decision-making About Upper Extremity Vascularized Composite Allotransplantation: Development and Usability Study
Source: JMIR Form Res. 2023 Feb 7;7:e44144. doi: 10.2196/44144 (PMC9944141; doi:10.2196/44144)
Supplement: Multimedia Appendix 2 [file formative_v7i1e44144_app2.docx]

| **Supplementary File 2. Focus Group Website Materials and Associated Questions** | |
| --- | --- |
| **Website Materials Displayed** | **Questions Asked** |
|  | |
| ***Website Name*** | |
| Website name | What do you think of this name of the website? |
| ***Website Logo*** | |
| Displayed possible images of the logo | What do you think about [the logo]? |
| ***Mission, Vision, Purpose Statement*** | |
| Displayed an example statement. | What do you think of this text and how could we improve it?  What term should we use – Mission, Vision, or Purpose Statement? |
| ***Website Site Map*** | |
| Displayed the main categories of the website and the subcategories under each.  *Example:*  What is VCA?  Definition  Status  History  Worldwide Performance  Functional Outcomes  Psychosocial Outcomes  Potential Benefits  Limitations  Unknowns  Myths and Facts | What do you think of the topics covered?  Are the topics in the right order?  What topics should we add? What topics should we delete?  Can you think of better names for the topics?  [If participant recommends a new topic]:  What information should be covered in each section? |
| ***Terminology Preferences*** | |
| VCA | Should we use the acronym “VCA” (Vascularized Composite Allotransplantation) in the website?  Do people need to know about VCA as a category of organs (like hand, face, uterus, penis transplants)? |
| Hand/Arm Transplantation | What is the best way to refer to Upper Limb VCA?   1. Hand transplantation 2. Upper limb transplantation 3. Hand/arm transplantation 4. Your/my new hand 5. New limb |
| Graft Survival | Do people know what “graft” means?  What does “graft survival” mean to you?   - Website text example: “In a study reporting on upper limb transplantation outcomes and survival rates, there were no deaths in the 50 patients who received an upper limb transplant. In these 50 patients, the graft survival rate of their transplanted limbs was 90%.” |
| Evaluation | When you hear the phrase “transplant evaluation process,” what does “evaluation” mean to you? |
| Upper Limb Transplant Researchers | When referring to upper limb transplant researchers, which term should we use?   1. “Medical researchers” 2. “Scientists” 3. “Transplant researchers” 4. “Research doctors” |
| Prosthetic vs. Prosthesis | Which term do you like better?   1. “Prosthetic” or 2. “Prosthesis” |
| Anti-Rejection Medication | When we refer to the medicine that hand transplant recipients take to prevent rejection, what is the best term to use?   1. “Anti-rejection medication” 2. “Anti-rejection medicine” 3. “Immunosuppressants” |
| Transplant Success | When you hear the concept of “transplant success,” what does that mean to you?  Here are three ways one could interpret the concept:   1. Surgical success in terms of attaching the hand to the body 2. Hand function gained from the transplant 3. The relevant function gained from a transplant vs. a prosthetic   Which interpretation fits your idea of success better?  Do you have other ideas for what success means? What counts as success? |
| ***Myth/Fact Section*** | |
| Displayed screenshot of 18 myth/facts.  Example:  “MYTH: I do not need hand therapy to get good function back.  FACT: The amount of functional ability patients get out of their transplanted hand or upper limb is related to their active participation in and adherence to the hand therapy program.” | What do you think of this section?  How can we improve it? What needs to be changed?  Do the answers sound appropriate?  What other myths should we add?  Do you like the myth/fact format? Or would you prefer a Frequently Asked Questions format? |
| ***Data Tables & Graphics*** | |
| 1. Number of hand/arm transplants performed in the US and Worldwide 2. Number of hand/arm transplant recipients by gender 3. Number of hand/arm transplant recipients by race/ethnicity 4. Number of hand/arm transplant recipients by age 5. Number of hand/arm transplants performed in the US by hospital 6. Map of active hand/arm transplant centers in the US 7. Public willingness to donate VCA organs 8. Willingness to donate one’s own hands vs. their family’s hands 9. Evaluation process diagram 10. Historical timeline of hand/arm transplants 11. Average Short-Form 36 scores for hand transplant recipients and prosthesis users 12. Patient evaluation for hand/arm transplant program at one transplant center 13. Estimated costs for hand/arm transplantation and prosthesis adoption in the US 14. Rejection rate comparisons for psychosocial predictors of hand/arm transplantation 15. Inclusion and exclusion criteria for hand transplant recipients used by several USA hand transplant centers | Can you please tell us what the table means to you?  How can we improve the table/graphic?  What other data tables should we add? What would you like to know? |
| ***Photographs*** | |
| Photographs of hand/arm transplant recipients showcasing the appearance of transplanted limbs and functional capabilities.  Photographs of military service members performing tasks with prosthetic devices.  Photographs of new types of prostheses, including different types of myoelectric prostheses.  Photographs of hand/arm transplant recipients participating in hand therapy. | Which photo(s) do you like best?  What other pictures or images should we put on the website?  Do you prefer photographs or technical drawings? |
| ***Website Screenshots*** | |
| Home landing page with website logo, site map section headers, pictures of hand/arm transplant recipients, and purpose statement.  Content page with sub-section written text providing educational content interspersed with video testimonies from hand/arm transplant recipients and providers. | What are your impressions of these website drafts? Do they look in logical order?  How can we make the website look like it is made especially for people with upper limb amputations?  How can we make the website more sensitive to the needs of people with upper limb amputations – in terms of using the website, and in the content and design.  How can we make the website more sensitive to the needs of people of diverse ethnic and racial backgrounds?  How could we make the language sound more empowering to people with upper limb amputations?  What would help encourage people with upper limb amputations to go to the website?  What makes a website easy for you to navigate? What features does it have?  Comprehensibility  What phrases were hard to understand?  How can we make it easier to understand?  Is there anything we could get rid of? |
| ***VCA Recipient Quotes*** | |
| Displayed 8 hand/arm transplant recipient quotes regarding the pros and cons of hand/arm transplantation. | Which quotes do you like the best?  Are there any quotes we should cut? |
