# Supplementary figures and images for: A Patient-Centered Website (Within Reach) to Foster Informed Decision-making About Upper Extremity Vascularized Composite Allotransplantation: Development and Usability Study
Source: JMIR Form Res. 2023 Feb 7;7:e44144. doi: 10.2196/44144 (PMC9944141; doi:10.2196/44144)

## Slide 1
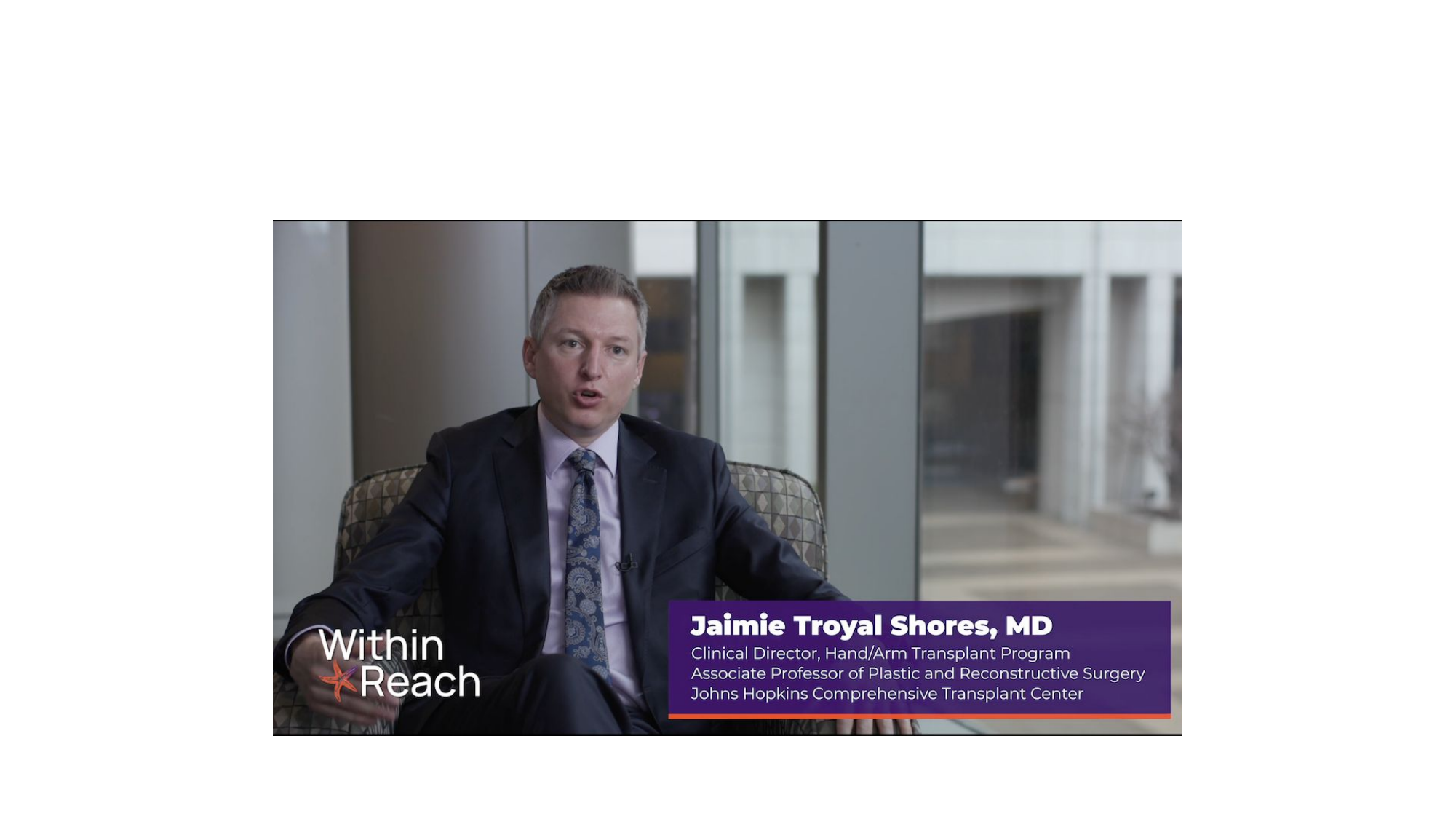

Supplement: Multimedia Appendix 3 [file formative_v7i1e44144_app3.pptx]

## Slide 1
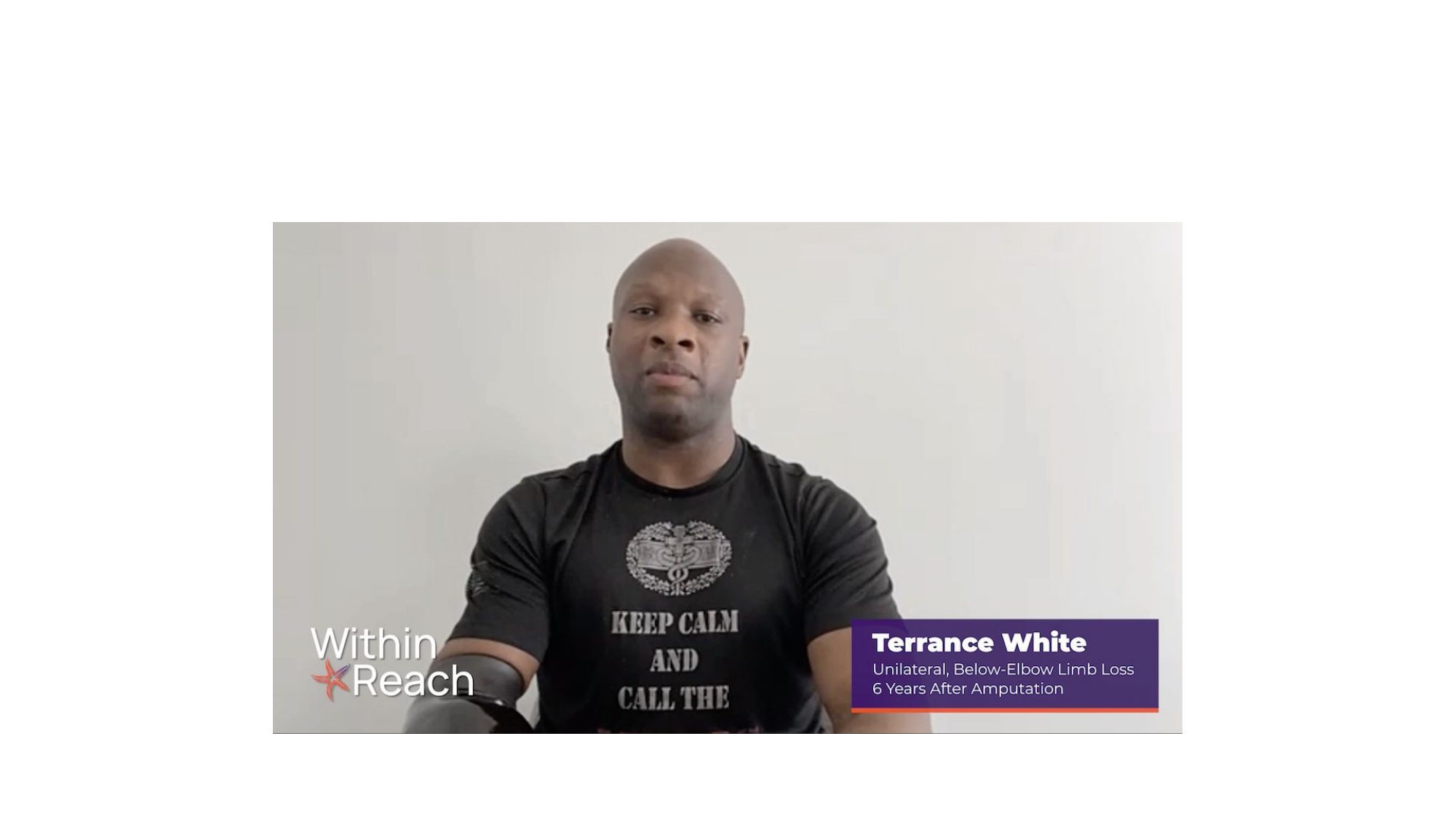

Supplement: Multimedia Appendix 4 [file formative_v7i1e44144_app4.pptx]

## Slide 1
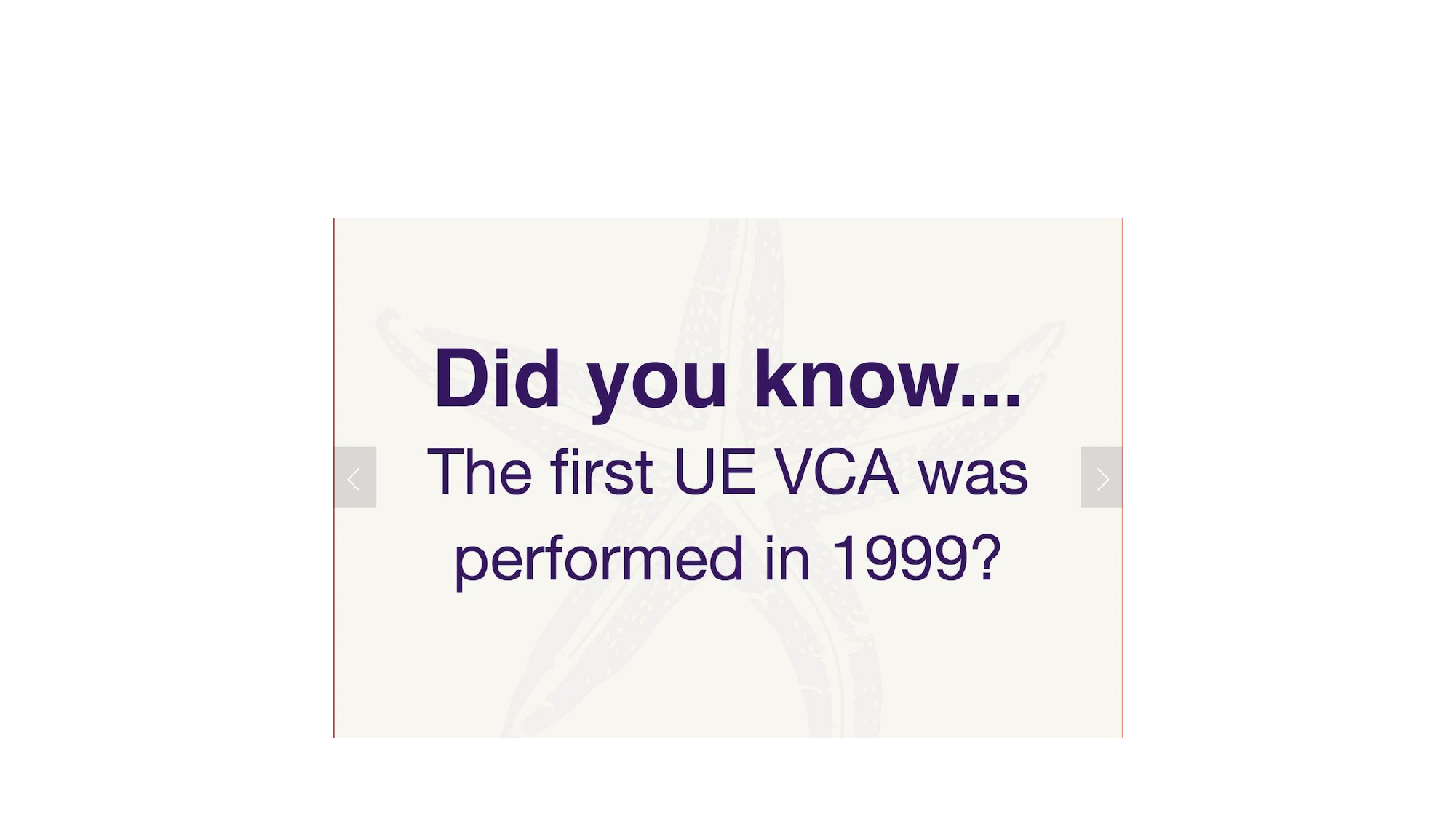

Supplement: Multimedia Appendix 5 [file formative_v7i1e44144_app5.pptx]

## Slide 1
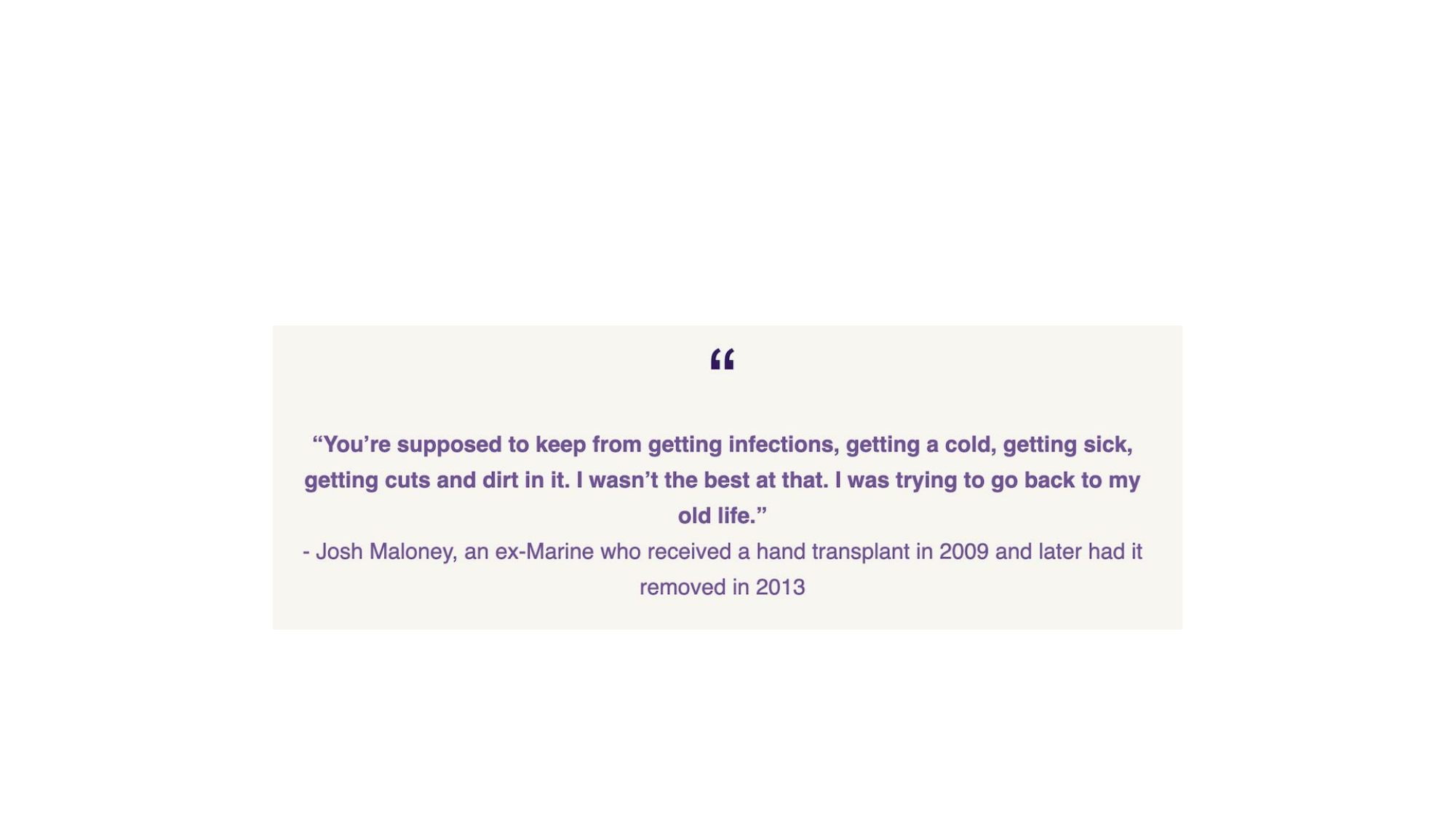

Supplement: Multimedia Appendix 6 [file formative_v7i1e44144_app6.pptx]

## Slide 1
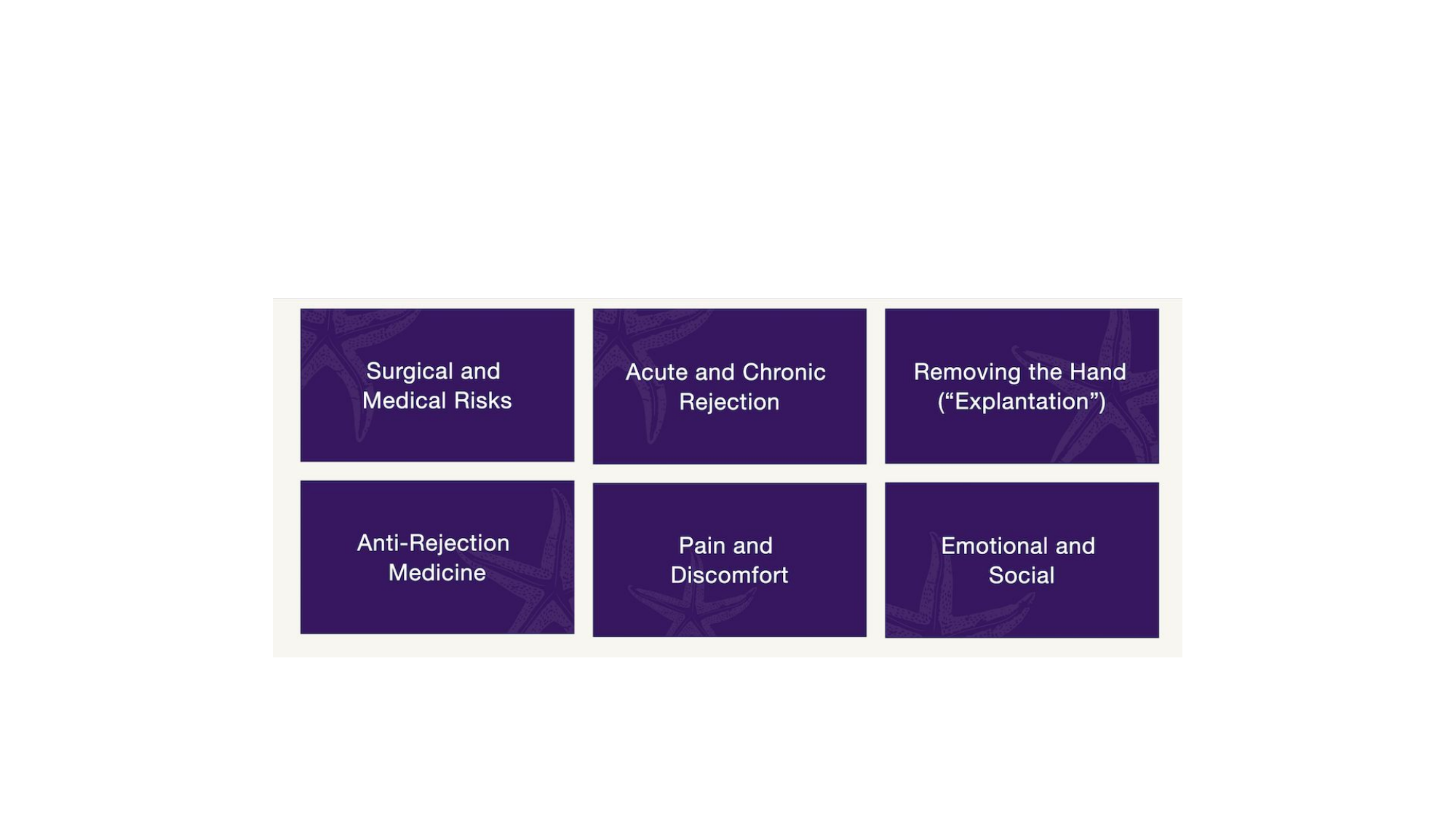

Supplement: Multimedia Appendix 7 [file formative_v7i1e44144_app7.pptx]

## Slide 1
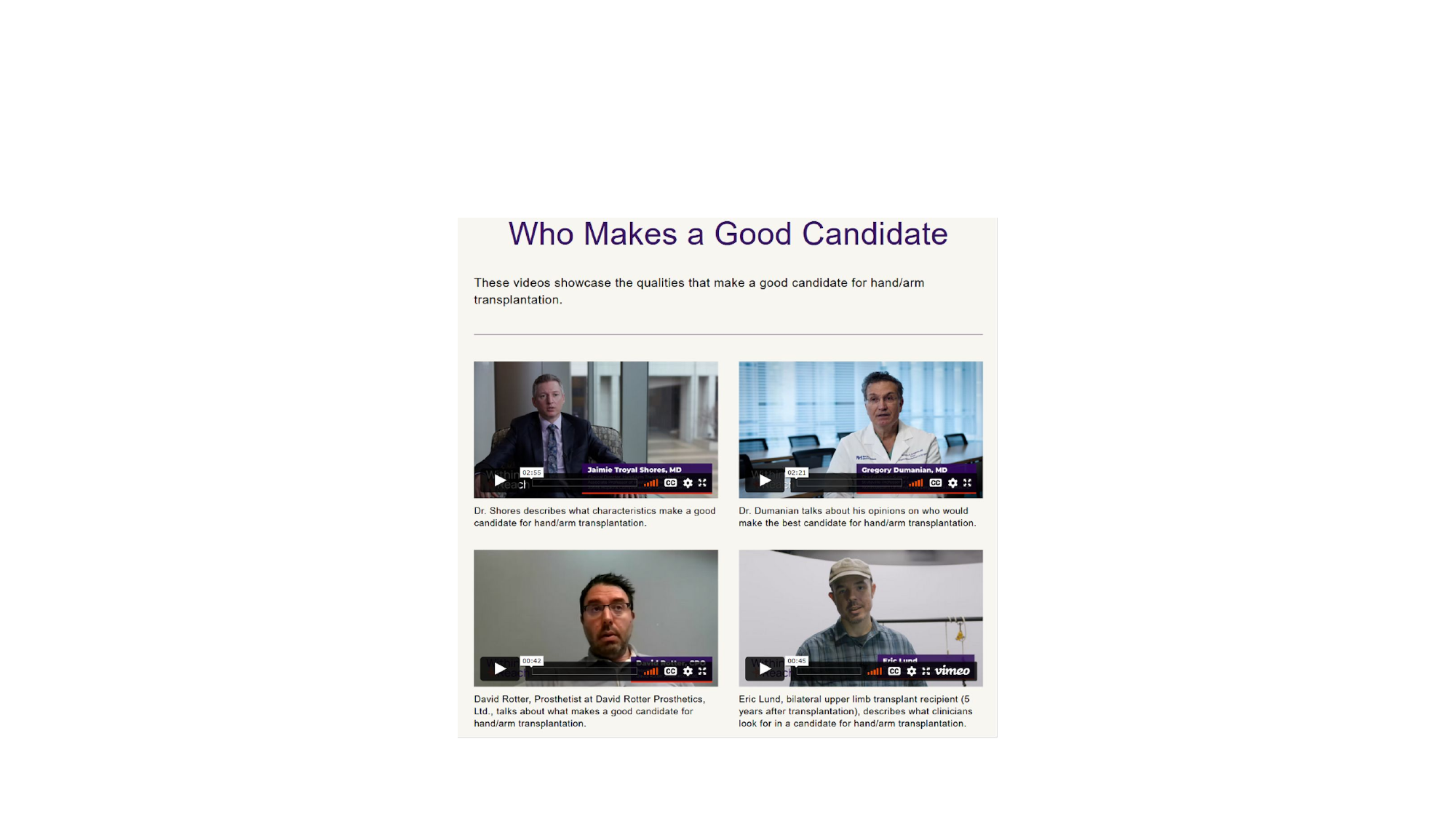

Supplement: Multimedia Appendix 8 [file formative_v7i1e44144_app8.pptx]
